# Component library creation and pixel array generation with micromilled droplet microfluidics

**DOI:** 10.1038/s41378-024-00839-6

**Published:** 2025-01-14

**Authors:** David McIntyre, Diana Arguijo, Kaede Kawata, Douglas Densmore

**Affiliations:** 1 Biomedical Engineering Department, Boston University, Boston, MA USA; 2 Biological Design Center, Boston University, Boston, MA USA; 3 Electrical and Computer Engineering Department, Boston University, Boston, MA USA

**Keywords:** Engineering, Nanoscale devices

## Abstract

Droplet microfluidics enable high-throughput screening, sequencing, and formulation of biological and chemical systems at the microscale. Such devices are generally fabricated in a soft polymer such as polydimethylsiloxane (PDMS). However, developing design masks for PDMS devices can be a slow and expensive process, requiring an internal cleanroom facility or using an external vendor. Here, we present the first complete droplet-based component library using low-cost rapid prototyping and electrode integration. This fabrication method for droplet microfluidic devices costs less than $12 per device and a full design-build-test cycle can be completed within a day. Discrete microfluidic components for droplet generation, re-injection, picoinjection, anchoring, fluorescence sensing, and sorting were built and characterized. These devices are biocompatible, low-cost, and high-throughput. To show its ability to perform multistep workflows, these components were used to assemble droplet “pixel" arrays, where droplets were generated, sensed, sorted, and anchored onto a grid to produce images.

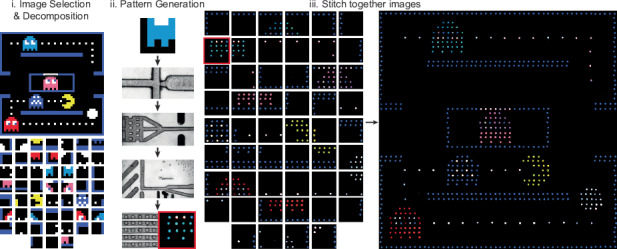

## Introduction

Droplet microfluidics present a high-throughput method for biological and chemical screening at the single-cell level. Platforms using droplet microfluidic technology have made fundamental advancements in protein engineering^1^, single-cell sequencing^2^, single-molecule detection^3^, and nanoparticle synthesis^4^. Despite these advances, the use of droplet microfluidics has been limited primarily to niche applications in commercial devices or use by experts^5^. One major barrier to microfluidic adoption is the dependence on lithographic fabrication techniques to make PDMS devices. While the surface properties and fabrication resolution make such devices ideal for most applications, the slow design cycle and need for a clean room have limited widespread use. Furthermore, the manual processes of pouring, curing, punching holes, and bonding a device make PDMS devices difficult to scale, limiting their commercial potential^6^. Prototyping microfluidic devices directly in commercially relevant thermoplastics would reduce substrate transfer issues during scale-up, reducing the time and cost of an individual design.

Laser cutting^7^, 3D printing^8^, and micromilling^9^ have all been implemented as rapid prototyping methods for microfluidic devices. Laser cutting is an established method for microfluidic fabrication, where patterns are directly etched into a plastic, adhesive, or paper substrate with a CO_2_ laser^10,11^. While common methods can create features as small as 100 *μ*m, laser cutting with standard setups produces channels with a pointed cross-section, preventing accurate transfer to other fabrication methods or the use of geometries with multiple depths. Furthermore, laser cutting is not compatible with some thermoplastics (e.g. polycarbonate, polyvinyl chloride) and has been shown to inhibit biological reactions due to the material leaching caused by the high-intensity laser^12^.

Recently, 3D printed microfluidics has garnered significant interest^8^. Common 3D-printing techniques such as stereolithography, multi-jet modeling, and fused deposition modeling, have been shown to produce features 100 *μ*m^13^, 750 *μ*m^14^, and 350 *μ*m in size^15^, respectively. In stereolithography only photo-curable substrates are used, limiting scalability to more common mass-produced substrates. The current printing resolution, surface properties, and device transparency impede the adoption of 3D-printed droplet microfluidics for biological high-throughput applications^16^.

Here, we show the creation and application of a droplet microfluidic component library with rapid prototyping. By combining micromilling and conductive ink electrode integration^17,18^, components for droplet generation, reinjection, anchoring, picoinjection, coalescence, and fluorescence-activated droplet sorting were developed. Micromilled droplet microfluidics present an alternative to PDMS devices, either as a direct replacement or as a companion to quickly iterate through initial designs. We show the library’s ability to perform multistep workflows by conducting fluorescent cell sorting and pixel array generation.

The ability to have quality control and testing methods that are quickly both verifiable and serve as a diagnostic tool in the case of component failure are extremely valuable. Our pixel array provides both methods. It creates a visually verifiable “signature” that the upstream droplet processing is correct. This is verifiable by the human eye once the images have been processed or more importantly long-term by machine vision software. This technique provides “pixel-level” accuracy which leads to the second aspect of diagnostic ability. By understanding which droplet(s) are incorrect, you can directly track the droplets to their place in the upstream workflow and identify failed experiments, bad components, or ineffective sensing thresholds.

## Results

### Development and characterization of a micromilled droplet microfluidic component library

**Fig. 1:**
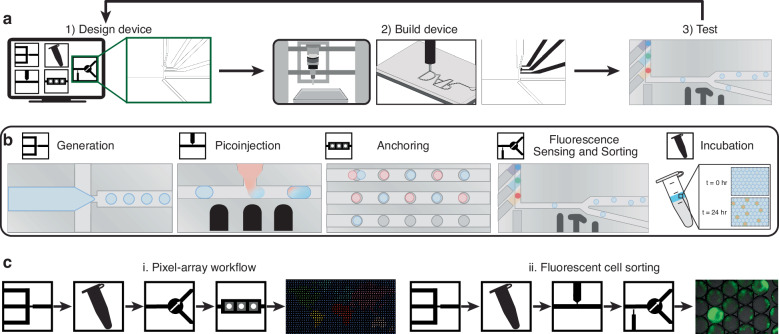
Overview of fabrication techniques and component library. **a** Design, build, test cycle for droplet microfluidics. **b** Droplet component library including droplet generation, anchoring, incubation, picoinjection, and fluorescence sorting. **c** Example droplet microfluidic workflows. Droplets are generated, reinjected, sensed, sorted, and anchored in a pixel array. Additionally, droplets with cells are generated, reinjected, picoinjected, sensed, and sorted for fluorescent cell sorting workflow

Most applications using droplet microfluidics require different configurations of common components. Such components encapsulate reagents, chemicals, or organisms into a droplet (droplet generation), sequentially insert more reagents into a passing droplet (droplet picoinjection), trap a droplet temporarily or permanently (droplet anchoring), measure the activity within a droplet (fluorescence sensing), or isolate droplets with a target behavior (droplet sorting)^19^. Exploiting the rapid design cycle (Fig. 1a), a full droplet component library was developed using desktop micromilling^17^ and conductive ink electrodes^18^ (Fig. 1b). This method of building sophisticated droplet microfluidic devices costs less than $12 per device and a full design-build-test cycle can be completed within a day (see Supplementary Fig. 1 for more detail on timing). The design cycle speed and reduced costs make polycarbonate micromilled devices an attractive alternative to PDMS or other rapid fabrication workflows. These devices can also be modeled with computer-aided design (CAD) software^20^.

#### Droplet generation

Droplet generation is the necessary component for droplet microfluidics. Through the controlled flow of two immiscible fluids (typically discrete aqueous phase and continuous oil phase), mono-disperse emulsions can be generated at high throughput. Previous work by our group has shown droplet generation with micromilled microfluidics with mineral oil^17,21^, as well as using this platform to generate a large dataset to build a machine learning-based design automation tool for single and double emulsion droplet generators^22–24^.

**Fig. 2:**
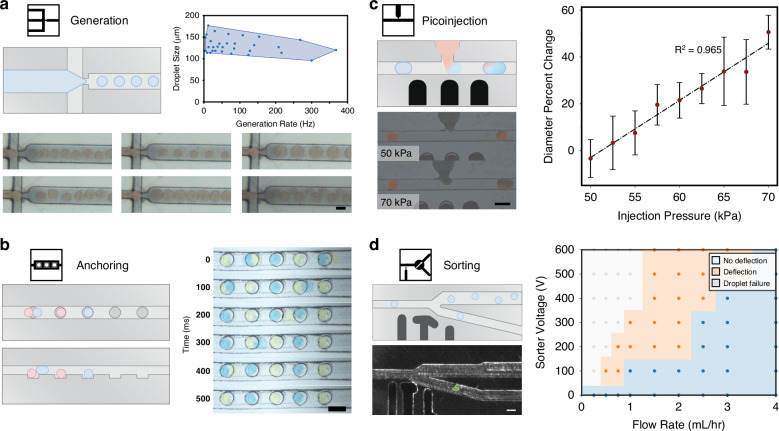
Characterization of droplet microfluidic components. **a** Monodisperse droplets are generated within a micromilled microfluidic device using an aqueous inner solution and outer oil. Using a single droplet generator, a wide range of droplet sizes and rates are produced. Different generator ranges are possible by changing device geometry or fluid properties. **b** Droplet anchoring with micromilled microfluidics. At a correct flow rate and anchor-droplet size ratio, droplets are transiently trapped at the anchor until disturbed by an additional droplet. Anchors were organized in a serpentine channel to produce a large droplet array, where droplets are spatially anchored and knocked to the subsequent anchor upon the introduction of another droplet into the serpentine. **c** Active electrode integration for picoinjection of two fluids within a microfluidic device. A similar phenomenon to coalescence is exploited between a continuous fluidic reservoir that can be changed to tune the amount injected, which has been shown to inject between −3% (taking fluid out of the droplet) and 50% of droplet volume within the same device and flow rates. **d** A high-voltage signal was selectively delivered to a single electrode for droplet sorting. Droplet sorting is sensitive to the flow rate of the droplet and applied voltage, generating too little force for deflection, successful deflection, or so much force that the droplet is stuck at the electrode and coalesces with other passing droplets in the device. Scale bars are 150 *μ*m

For most biological applications, fluorinated oils are preferred over mineral oil as they are insoluble to organic material and have a high oxygen carrying capacity and therefore are suitable for in-droplet cell culture^25^. As polycarbonate is less hydrophobic than PDMS (*θ*_*waterPC*_ ≈ 70°, *θ*_*waterPDMS*_ > 100°)^26,27^, droplet generation with fluorinated oils require channel surface treatment to make them hydrophobic. After hydrophobic surface treatment with Aquapel, a micromilled droplet generator with a 75 *μ*m wide orifice generated monodisperse droplets 95 *μ*m to 176 *μ*m in diameter at rates between 4 and 368 Hz (Fig. 2a). Flow rates ranged from 4.16 to 166.7 *μ*L/min and 0.2 to 16.66 *μ*L/min for oil and water, respectively. Generation below the lowest flow rates used did not produce droplets at a constant rate or size. Generation above the flow rates used broke the device’s adhesive bonding or produced polydisperse droplet doublets.

#### Droplet anchoring

Once generated, droplets may need to be stored off-chip, incubated at specific temperatures, and reinjected into a device for downstream analysis. Droplet incubation is essential to grow monoclonal cell cultures or for reactions to occur. On-chip delay of droplets allows for incubation at a set time and at a set temperature in a way that is observable under a microscope and where a droplet is fixed spatially. Effective droplet trapping is needed for long-term imaging of a droplet, essential to study temporal changes in cell growth or genetic circuit activity^28,29^.

To trap and incubate droplets on-chip, microfluidic anchors were created, guided by previous work in PDMS devices (Fig. 2b and Supplemental Video 1)^30^. By using channel depths shallower than the droplet diameter, droplets are squeezed into a non-spherical shape which increases the droplet/oil interface surface energy and hydrodynamic resistance in the channel. By adding an area of differential depth into the device, the droplet becomes “stuck" at this interface as the droplet is less squeezed and in a more energetically favorable state. At the correct flow rates, this produces anchors that trap droplets at this interface as flowing oil passes around. Droplets are sequentially knocked down an array of anchors as the upstream droplet takes its place. If the flow rate is too fast, the droplet passes right by the anchor and is not trapped. If the flow rate is too slow, the droplet is permanently stuck in the anchor and either blocks another droplet from passing or lets the droplet pass over the anchor without deflection. These anchors can be placed within a large serpentine to build a droplet anchor array, where droplets are spatially controlled by determining the number of droplets that are deflected after it (Fig. 2b). Anchor creation is much simpler with micromilling than PDMS: channels at multiple depths are created by changing the design which is translated to machine instructions in contrast to the multiple masks needed for multi-layer PDMS^31^.

#### Picoinjection

A basic operation of molecular biology or chemistry protocols is the liquid handling addition of reagents at accurate volumes and times. Therefore, the translation of these protocols into microfluidics requires a component for controlled insertion into droplets. Fluids are directly added to a droplet with picoinjection, where fluid from a reservoir held at a slightly positive pressure differential is injected into a passing droplet^32^. Upon contact between the droplet and reservoir, an interruption to the stabilizing forces from surface tension is needed to transiently connect the two fluid bodies. This can be done passively through device geometry^33^ or actively with a high-voltage electric field^32^. Picoinjection volume has been shown to be accurate from droplet to droplet, and injection volume can be tuned by changing the injection pressure, droplet velocity, or applied voltage.

Micromilled droplet picoinjectors were developed using conductive ink electrodes to produce a wide range of injection volumes by tuning droplet velocity, input droplet size, input voltage, and injection pressure (Fig. 2c). By changing injection pressure during picoinjection at approximately 100 Hz, the average change in droplet volume from injection was varied from −3% (pressure was below equilibrium) to +50% (Fig. 2c). Data was analyzed from one second of video for each pressure. This control over injection allows for the user to treat picoinjection as they would any pipetting step: if the injection amount is known, the concentration of the reservoir can be set accordingly to accurately perform multi-step or time-sensitive protocols within droplets.

#### Fluorescence sensing

Methods to measure a phenotype are necessary for high-throughput screening of biological systems. Adapting a previously developed fiber-optics-based system^34^, a multi-channel fluorescence sensor was created for micromilled microfluidics (Supplementary Fig. 2). In this system, 50 mW lasers (OptoEngine) are coupled to 200 *μ*m fibers and inserted into channels. As polycarbonate is rigid, there is less concern for device tearing during fiber insertion as there is with PDMS. These channels are inserted at an angle to a channel where a droplet passes through and makes a 90° turn. This orientation allows for a single 200 *μ*m photomultiplier tube (PMT) fiber to collect excitation from 3 different lasers while minimizing background noise. The signal from all three lasers is captured by the PMT fiber. Background signal and light from the lasers is filtered out with two multi-bandpass filters (446/523/600/677 nm; 25 nm bands; Semrock). All optics, lasers, and connectors operate within a closed system to minimize noise and limit safety issues from exposed laser beam paths.

Once captured by the PMT, filtered light is converted to an analog voltage signal and digitized by a microcontroller (Arduino). An algorithm is run in real-time on the microcontroller to identify peaks and their magnitude. This is performed by first recording a moving average of the baseline data points. Data points classified as a peak are not added to this baseline to avoid drift. Once the signal exceeds a threshold (set as a multiple of the standard deviation of the moving average), the signal is classified as a peak. Peak recording continues until the signal returns back below the threshold value. If multiple excitation channels are used, peaks occurring within a set group time are collected together to give a 3-dimensional fluorescent signature for a single droplet. These results can then be compared against a target value and trigger an electronic actuator if downstream steps are needed.

**Fig. 3:**
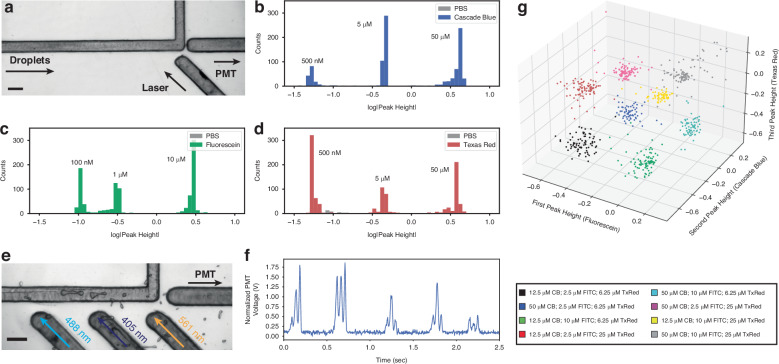
Characterization of droplet microfluidic fluorescence sensing. **a** Single-channel fluorescent sensing was performed within a micromilled device. Lasers of a specific wavelength are inserted into a microfluidic device to excite a fluorescent moiety inside of a droplet. Emission is then captured by a second fiber connected to a PMT. Scale bar is 200 *μ*m. A mixed population of droplets containing Cascade Blue (CB), Fluorescein (FITC), and Texas Red (TxRed) fluorophores were inserted into this system and excited by 405 nm, 488 nm, and 561 nm lasers, respectively. By detecting three distinct populations and no negligible background peaks from PBS droplets, this fluorescent system can detect levels at a minimum of (**b**) 500 nM Cascade Blue, (**c**) 100 nM Fluorescein, (**d**) and 500 nM Texas Red. The scale bar is 200 *μ*m. **e** 3-channel fluorescent detection. Three excitation fibers are inserted into the device and collected with a single emission fiber inserted at a channel right angle turn to detect excitation from each laser. **f** After excitation with each laser, a three-peak signal is acquired per droplet. This signal is then collected by a microcontroller and peaks are automatically detected. **g** Multichannel fluorescent measurement of a mixed population of eight unique groups of droplets are measured and grouped by k-means clustering. Reliable detection of droplets using fluorescence is crucial for coupling with other devices for isolation and biological integration

To validate this custom fluorescent detection system, the limit-of-detection was found for individual excitation channels using a device with a single excitation fiber (Fig. 3a–d and Supplemental Video 3). For the single channel measurements, an additional channel-specific bandpass filter was added in front of the PMT to further eliminate stray light (460-60 BFP Filter; 525-39 GFP Filter; 630-60 TXRed Filter; Thorlabs). Using 405 nm, 488 nm, and 561 nm lasers, three different fluorophore concentrations in 140 *μ*m droplets could be distinctly detected down to 500 nM Cascade Blue, 100 nM Fluorescein, and 500 nM Texas Red (Fig. 3b–d). Negligible peaks were detected with each laser when flowing through PBS droplets of the same size.

Next, these three channels were combined to detect 8 distinct populations of droplets. Using three spatially separate lasers and a single PMT, each droplet produced a 3-peak trace which is automatically detected and grouped into a single sample (Fig. 3f). These 8 separate groups were accurately detected with the fluorescent sensor and separated into distinct populations using k-means clustering (Fig. 3g). Accurate fluorescent sensing of droplets is essential for coupling to additional actuators for isolation and biological integration.

#### Droplet sorting

After generating and processing droplets, recovery of high-performing samples from the device may be needed. This is particularly important for biological analysis, where an observed phenotype can be linked to a genotype via downstream sequencing. Droplet sorting can be achieved passively via size-based selection^35^, or actively with electrical^36^, pneumatic^37^, acoustic^38^, or magnetic actuation^39^. Sorting depending on phenotype or droplet characteristics requires an upstream sensor that provides feedback to the actuator^40^.

The operating conditions for droplet sorting were characterized by changing the total flow rate and electrode voltage of the device (Fig. 2d). The high voltage signal was kept continuously on to see the effect over the entire droplet population. Through this, three behavior regimes were found: at high flow rates or low voltages, the generated force was too low and no deflection occurred; at a medium flow rate and higher voltages, droplets were deflected successfully into the sort channel; and at low flow rates and high voltages, the dielectrophoretic force immobilized a passing droplet at the electrode interface and led to uncontrolled coalescence with adjacent passing droplets (Fig. 2d and Supplemental Video 4). For example, at a flow rate of 1 mL/hr, sorter voltages below 100 V resulted in no deflection, while voltages above 400 V resulted in droplet failure. Droplets were selectively sorted with a user-defined waveform from a function generator pulsing a 1.2 kV, 45 kHz sine wave on and off, showing its potential for single droplet selection once connected with an upstream sensor. Fluorescent droplet sensing and sorting was performed at a maximum of 80 droplets per second.

#### Biocompatibility

The ability to measure the properties of single cells isolated from a large population is one of the main motivations of droplet microfluidics^41–43^. Single-cell encapsulation is necessary for scRNA-seq^44^, ensuring monoclonal cultures^45^, or screening through a combinatorial library^1^. Single-cell encapsulation into a droplet is a Poisson process as described in Supplementary Note 1^46^, where the Poisson distribution defines the probability of encapsulating single-cells in droplets from a diluted input culture.

**Fig. 4:**
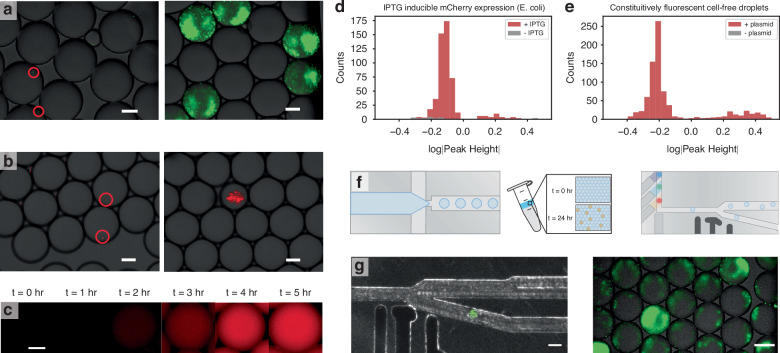
Measurement of biological expression levels with a micromilled fluorescent sensor. Single cells of (**a**) constitutive GFP expression in *E. coli* and (**b**) constitutive mRuby expression in *S. cerevisiae* are encapsulated within a microfluidic device. The scale bar is 100 *μ*m. **c** Cell-free TXTL mix with a plasmid constitutively expressing mCherry is encapsulated within a droplet and incubated over 5 hours until significant expression is observed. The scale bar is 50 *μ*m. **d**, **e** Measurement of biological expression levels with a micromilled fluorescent sensor. Using a 561 nm laser, fluorescent expression of (**d**) IPTG-induced mCherry expression in *E. coli* and (**e**) constitutive mCherry expression in TXTL mix. Negligible peaks were measured in blank droplets. **f** Schematic for single-cell droplet generation, overnight incubation, and fluorescence sensing. **g** Droplet sorting of IPTG-induced GFP expression in *E. coli*. The scale bar is 100 *μ*m

Micromilled droplet generators were used for single-encapsulation of *E. coli* constitutively expressing GFP and *S. cerevisiae* constitutively expressing mRuby (Fig. 4a–b). Cell concentrations (*cells*/*μL*) in a sample prior to dilution were found by converting Optical Density (OD) to cell number for *E. coli* ($2.88* 1{0}^{8}\frac{cells}{mL* OD}$) and *S. cerevisiae* ($1* 1{0}^{7}\frac{cells}{mL* OD}$). Each of these species was encapsulated at the single-cell level within droplets, and shown to match the Poisson distribution (Fig. 4a–b; Fig. S3). To show droplet biocompatibility and amplify the fluorescent signal, droplets containing single *E. coli* and *S. cerevisiae* cells were incubated overnight at 37 °C and 30 °C, respectively. A water bath was added inside the incubator to saturate humidity and prevent evaporation. In both cases, cell populations grew from a single cell within droplets (Fig. 4a–b). In the *S. cerevisiae* droplets, a significant change in droplet diameter was observed as cells grew, an expected occurrence due to glucose consumption which results in the osmotic effect causing water to leave the droplet.

Cell-free transcription-translation (TXTL) mix was also shown to be compatible with micromilled droplet microfluidics (New England Biosciences PureXpress). TXTL mix is an attractive alternative to whole-cell work for prototyping synthetic biology systems, eliminating the need for cell culture, inserting DNA into the cell, or toxicity concerns^47^. This is especially true for droplet microfluidics, where droplets containing a cell-free mix act as individual bioreactors without the need to encapsulate cells. TXTL mix is expensive if bought directly from a vendor (~ $1000 per mL), and therefore miniaturization of the sample into droplets is key for screening through a large number of samples in a cost-effective manner. In a micromilled droplet generator, TXTL mix was encapsulated with a set concentration of a constitutive mCherry expressing plasmid. After incubating at 37 °C for 5 hours, TXTL expressed observable mCherry within droplets (Fig. 4c).

The sensitivity of the custom microfluidic fluorescence detection system was evaluated against biological expression levels in both *E. coli* and TXTL mix. Upon excitation with a 561 nm laser, *E. coli* cultures with IPTG-inducible mCherry expression and TXTL mix constitutively expressing mCherry produced a significantly higher signal than uninduced *E. coli* cultures and blank TXTL mix droplets, respectively (Fig. 4d–e). In both cases, samples with fluorescent levels higher than the normal distribution of most of the population can be attributed to large droplets created from nonspecific coalescence during incubation.

Additionally, IPTG-inducible *E. coli* cells expressing GFP were encapsulated in droplets. These droplets were incubated at 37 °C overnight with a water bath and reinjected into a microfluidic device for fluorescence-activated droplet sorting (Fig. 4f). The droplets were sensed with the single-channel fluorescence sensor (488 nm laser) and sorted based on GFP output expression (Fig. 4g). This proof-of-concept shows the biocompatibility of multiple rapidly-prototyped microfluidic components in a multi-step workflow. To show the potential biological applications for the microfluidic library, Supplementary Note 3 details the methods for protein engineering screening and genetic circuit analysis.

### Programmable droplet pixel arrays

**Fig. 5:**
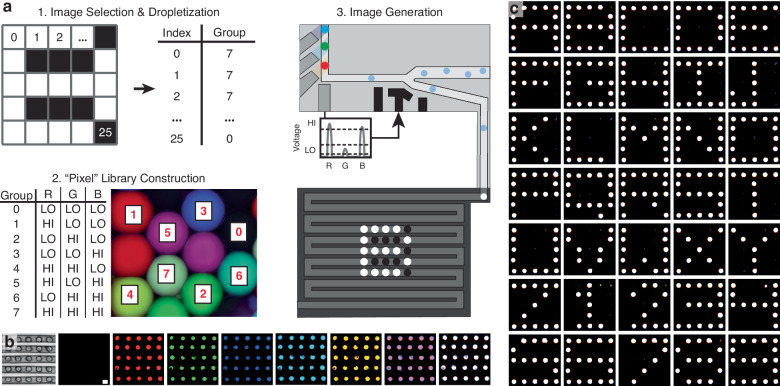
Workflow for droplet pixel array generation. **a** First, an image is selected and “dropletized" into 25 pixels. Each of those pixels are then grouped together into the closest color possible with a droplet. The closest color is defined by high and low levels of red, green, and blue. Next, a droplet pixel library is generated containing 8 colors total. These droplets are then inserted into a fluorescent sorting device, which sorts droplets in an order determined by a traversal of the droplet image. Sorted droplets are then anchored on the pixel array, and captured by a fluorescent scope. **b** Using this workflow, 8 distinct colors are generated, sensed, sorted, and anchored onto the pixel array. The scale bar is 150 *μ*m. **c** Using a two-color input, a black-and-white alphanumeric symbol library was generated on a droplet pixel array

As an example of using micromilled droplet microfluidics in multistep screening protocols, multiple components were combined to create programmable droplet pixel arrays (Fig. 5a). First, a multi-color droplet pixel library was constructed by combining high and low concentrations of blue, green, and red fluorophores. Next, five-by-five user input images were divided into a grid and fixed to the closest droplet color using an open-sourced software tool (https://github.com/CIDARLAB/drop2image, Supplementary Note 2, and Supplementary Video 5). This pattern is converted into a list of instructions, which is sent to a 3-channel fluorescent sorting and anchoring device. In the order given by the instructions, the device specifically selected the target droplet color, which was then captured onto a five-by-five droplet anchor array. In this array, droplets are sequentially knocked into the next anchor until they reach their final location. Once complete, the device stops sorting, and a final image recreation is captured with a camera connected to a fluorescent microscope (Nikon). This workflow requires a high level of control: if the resistance in the anchor array is too low, droplets will not deflect and get stuck within the anchors, or the sorting electrode will not be able to deflect the droplet; if the resistance is too high, droplets will not be trapped within the anchor at all and nonspecific sorting occurs.

With this multi-component system, 8 distinct colors were immobilized and visually observed within the array by instructing the system to continuously deflect droplets of a single classified group (Fig. 5b). The distinct visual differences between all eight colors indicate that more complex patterns can be generated and observed on the droplet pixel array. To show the programmability of these pixel arrays, 35 unique instructions were sent to the microfluidic device to generate a 5 × 5 collection of black and white letters and numbers (Fig. 5c). This shows the accuracy of our system: in contrast to single color sorting, these symbols could only be generated if all 25 droplets were deflected in the exact fashion as the instructions. If an error occurs due to nonspecific selection, skipping over an anchor, or two droplets being stuck on an anchor, the symbol is incomprehensible.

**Fig. 6:**
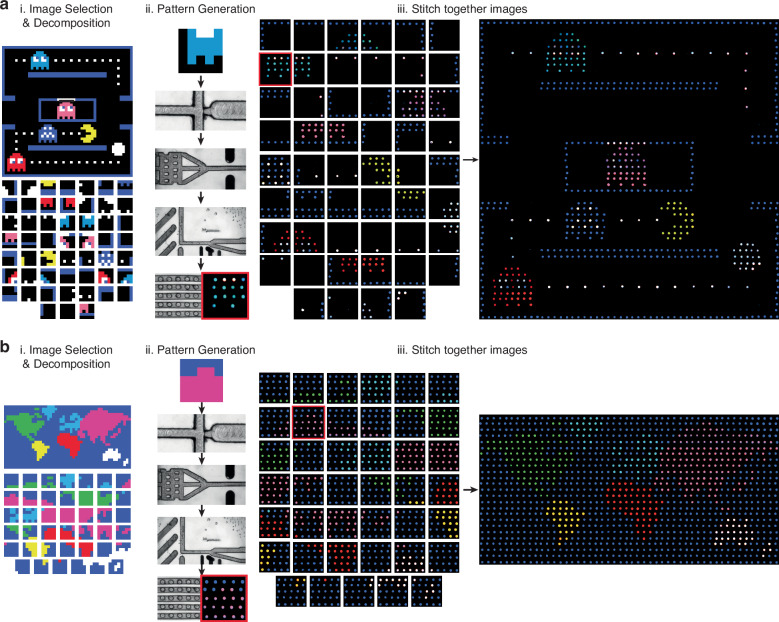
Large mosaic images are generated with a software-guided droplet pixel array. (i) A selected image is provided by the user and then decomposed into all of the unique 5 × 5 patterns present in the image. (ii) One by one, these patterns are generated by the droplet microfluidic workflow. (iii) Generated images are then stitched together to create large, 8-color images such as an (**a**) arcade game or (**b**) world map. Red square tracks pixel array across ii-iii

To further show the versatility and ability of the droplet pixel arrays to sense, sort, and anchor different droplet populations, large 8-color mosaic images were created using this system and a custom software tool. First, an image and mosaic size is specified, which is rendered into a large-scale pixel array. Next, this image is uploaded into an editable interface, where small edits and error corrections are made to the pixelated image. This image is then divided into 5 × 5 patterns that are generated by our microfluidic workflow. All unique patterns from this decomposition are identified, generated experimentally, and subsequently stitched together into a single image. This workflow was used to generate a 50 × 50 arcade game image. This image was decomposed into 100 different 5 × 5 grids (Fig. 6ai). Out of these, 52 unique patterns were sent to the microfluidic system, where an 8-color droplet population was generated, reinjected, subject to FADS, and anchored within the pixel array to create each pattern out of droplets (Fig. 6aii). These generated patterns are then stitched back together to produce a multi-color, large-scale pixel mosaic (Fig. 6aiii). The pixel-level accuracy of the array provides a visually verifiable signature that all upstream droplet processing is correct. This proof-of-concept shows the utility of desktop micromilled microfluidic in complex, multistep protocols.

## Discussion

Here, we demonstrated the development and characterization of a micromilled droplet microfluidic component library for droplet generation, re-injection, anchoring, picoinjection, fluorescence sensing, and sorting. These devices are biocompatible and are shown to be sensitive enough for fluorescence-activated droplet sorting of biological expression levels. To show the versatility of micromilled droplet microfluidics, these components are used to generate droplet pixel arrays for image production in a multi-step workflow. This workflow requires a high level of control: if the resistance in the anchor array is too high, droplets will not deflect and get stuck within the anchors, or the sorting electrode will not be able to deflect the droplet; if the resistance is too low, droplets will not be trapped within the anchor at all and nonspecific sorting occurs. Due to this high sensitivity, larger anchor arrays were limited by the resistance changes caused by the number of droplets immobilized changing the flow rate within the array.

Droplet generation rate and reinjection rates are limited by the device’s adhesive bonding. Long-term picoinjection is challenging because of the variation in picoinjection pressure and overall fluctuations in the picoinjection device’s input and output pressures. The picoinjection accuracy is dependent on the steady and periodic reinjection of droplets which can be affected by air bubbles in the inlets and outlets. The droplet throughput of fluorescence sensing and sorting is limited by the data collection and analysis speed of the microcontroller. This limitation is important for sorting droplets with cells due to the finite lifetime of the cell’s fluorescent output. Additionally, if picoinjection is added to the pipeline, the limited droplet throughput also adds hours to the workflow. Overall, optimization between flow rates, laser intensity, and delay between sensing and sorting actuation affects droplet reinjection periodicity, fluorescence sensing thresholds between droplet populations, and successful sorting and anchoring. This optimization between different parameters can be time and resource-intensive for applications involving fluorescence sensing, droplet sorting, or droplet anchoring. While manual optimization can be time-consuming, automation could be leveraged to improve device testing.

Although the cost and time advantages of micromilled microfluidics are immense, limitations prevent its complete replacement of PDMS devices for prototyping purposes. The CNC mill used in this study has a minimum channel dimension of 75 *μ*m, which prevents generating droplets smaller than this minimum dimension, inhibits performance at extremely high throughput, and limits how close excitation and emission sources are to the fluidic channels. While other mills have been shown to etch features as small as 600 nm into thermoplastics and metals, these present high infrastructure costs similar to clean-room fabrication^9^.

As fluid dynamics can be scaled, micromilled devices can be used as a rapid way to prototype completely novel designs. Additionally, if the goal of device development is to eventually port the system into a mass-manufactured thermoplastic, polycarbonate presents a way to test the fluidics in a rigid geometry with similar surface properties.

Micromilled microfluidics can be particularly useful for large-scale dataset generation for the development of Computer-Aided Design (CAD) software. Eventually, we believe microfluidic CAD programs will allow for high-level functional descriptions to be synthesized into droplet operations (GENERATE, SORT, MERGE, INJECT, etc.), analogous to the synthesis of digital, CMOS-based electronics. While multiple microfluidic CAD tools have been developed^20,48,49^, most lack thorough experimental characterization or an established dataset for performance prediction. Previous work has shown the potential of leveraging datasets from micromilled microfluidics: combining a large-scale dataset from micromilling and a small-scale dataset from PDMS, design automation of both single and double emulsion droplet generators was achieved in a fluid-agnostic manner^22^. A similar workflow could be applied to all of the different components developed here, to accurately map flow conditions and design geometries to performance metrics such as sorting accuracy or volume picoinjected. Once achieved, the droplet operations could be composed to automatically design microfluidic components for complex workflows. These then could be attached to separate pixel arrays which could be used to identify the most functional pipelines. Depending on the results in the array, the same CAD software could modify the designs automatically to address shortcomings either with new components or different parameters.

## Conclusion

Here, we show the development, characterization, and application of a droplet microfluidic component library produced in a rapid and inexpensive fashion by desktop micromilling and conductive ink electrodes. A micromilled droplet generator with a 75 *μ*m wide orifice generated monodisperse droplets 95 *μ*m to 176 *μ*m in diameter at rates between 4 and 368 Hz. Droplets were picoinjected at 100 Hz with the average droplet volume varying from −3% to +50 %. Using three-channel fluorescence sensing, a mixed population of 8 droplet groups was measured. Droplets were sensed, sorted, and anchored in 100s of unique 5 × 5 grids to generate pixel arrays which verified the overall system functionality. These components are biocompatible, can be paired with sensors sensitive enough to measure biological expression levels, and have been shown to function in complex workflows such as image generation and verification with droplet pixel arrays.

## Method

### Fabrication and electrode integration of microfluidic devices

Droplet microfluidic devices were fabricated with low-cost desktop micromilling, as previously described (Figure S1)^17^. The control and flow layers of the microfluidic devices were milled using a low-cost desktop CNC micromill (Othermill/Bantam Tools) from polycarbonate sheets with a thickness of 3.10 mm (McMaster-Carr) with endmills sized between 75 *μ*m and 1/8" (Performance Micro Tool). Electrodes were integrated using a carbon-based conductive ink (Bare Conductive Inc) as previously described^18^. In brief, electrode geometries are etched into the microfluidic device during fabrication. Flow channel electrodes are covered with an adhesive tape, followed by the application of conductive ink to the electrode channels with a tissue wipe. Pins were inserted into the microfluidic device to connect the electrodes to external circuitry. Excess conductive ink is removed with 91% isopropyl alcohol and another tissue wipe. Devices were sealed using an 81 *μ*m thick adhesive seal (Adhesives Research ArCare 90445Q). The hydrophobicity of the microfluidic channels was increased via surface treatment with Aquapel to prevent droplet coalescence within the device.

### Microfluidic operation

Droplet microfluidic components were operated with syringe pumps (Harvard Apparatus) or pressure pumps (Fluigent FlowEZ; Fluigent LineUp Push-Pull) connected to flow sensors (Fluigent Flow Unit M). Images were captured using a high-speed camera (IDT X-Stream) attached to an inverted microscope (Zeiss Axiovert 200 M) and an 18,000-lumen LED light source (Expert Digital Imaging). Images for fluorescence microfluidic devices were captured using a high-speed camera (pico.edge 4.2) attached to a fluorescence microscope (Nikon Eclipse Ti2-E).

All droplets were generated with Droplet Generation Oil containing a proprietary surfactant (Biorad) using a droplet generator design and flow rates as specified by the DAFD CAD tool (Fig. S5)^23^. After generation, droplets were stored off-chip by surface treating all tubing and tube walls with Aquapel and HFE-7500 (3M Novec 7500 Engineered Fluid) oil passed through an 0.45 *μ*m filter (Millipore Sigma) to limit droplet adhesion to the container. Once treated, droplets are stable off-chip for multiple days (Fig. S6A). If incubation is needed, evaporation of droplets is minimized by covering collected droplets with their aqueous phase fluid (water, media, etc.) as well as placing a water bath in an incubator to increase humidity. Droplets are reinjected via aspiration from their container into surface-treated tubing with a syringe/pressure pump (Fig. S6B). An air bubble is added prior to droplet aspiration to prevent droplet diffusion into the rest of the syringe fluid and ensure close packing of droplets for reinjection.

Design specifications for the droplet picoinjector and sorter used are described in Figs. S7 and S8, respectively. Delivery of a signal to the microfluidic device for droplet sorting or picoinjection was performed as previously described^18^. In brief, a 1.2 kV, 45 kHz sine wave was delivered to the device, either pulsed at 1 kHz to overcome stabilizing forces from surface tension between a passing droplet and reservoir for picoinjection or selectively actuated to exhibit a dielectrophoeretic force for droplet sorting. The electric field produced by this electrode was generated by placing ground electrodes in close proximity to the signal electrode. Additionally, electrode shields are placed around the reinjection area to prevent nonspecific droplet coalescence.

### Fluorescence detection

To deliver the excitation signal to the droplet, 50 mW lasers (405/488/561 nm; OptoEngine) are coupled to 200 *μ*m fibers (Thor Labs FT200EMT; Figs. S2 and S9). A photomultiplier tube (Thor Labs PMM02) attached to another 200 *μ*m fiber was used to collect the excitation signal from the droplet. Background signal and light from the lasers are filtered out with two multi-bandpass filters (446/523/600/677 nm; 25 nm bands; Semrock). For single channel measurements, an additional channel-specific bandpass filter was added in front of the PMT to further eliminate stray light (460-60 BFP Filter; 525-39 GFP Filter; 630-60 TXRed Filter; Thorlabs).

### Pixel array generation

All code to generate pixel array designs and operate the pixel array is available on GitHub (https://github.com/CIDARLAB/drop2image; Figure S4). An image is selected and split into 5 × 5 pixel arrays. Each image is “dropletized” based on the closest color in the droplet population. Next, a droplet pixel library is created by pooling together 8 separately generated droplet populations of varying levels of Fluorescein, Texas-Red, and Cascade Blue within the droplet. These droplets are then injected into a microfluidic fluorescence sorting device, which sorts droplets in the order determined by a traversal of the droplet image. The anchor array used for the generation of the 5 × 5 pixel array is described in Figure S10. An Arduino Uno microcontroller converts the analog PMT signal to a digital signal for the upconverter circuit.

## Supplementary information

Supplemental Material

Experimental Planning Template Datasheet

Supplemental Material Videos
