# Supplementary material for: Component library creation and pixel array generation with micromilled droplet microfluidics
Source: Microsyst Nanoeng. 2025 Jan 14;11:6. doi: 10.1038/s41378-024-00839-6 (PMC11733136; doi:10.1038/s41378-024-00839-6)
Supplement: Supplementary file 1 — Supplemental Material [file 41378_2024_839_MOESM1_ESM.docx]

Supporting Information for

**Component library creation and pixel array generation with micromilled droplet microfluidics**

David McIntyre, Diana Arguijo, Kaede Kawata, and Douglas Densmore

Corresponding author: Douglas Densmore

E-mail: dougd@bu.edu

**Supplementary Notes:**

**Supplementary Note 1: Cell Encapsulation**

Generally, cell encapsulation into a droplet is a Poisson process^1^. The Poisson distribution is defined as:

$$P\left( k \right)= \frac{\lambda^{k}{exp}^{-\lambda}}{k!}$$

where $k$ is the number of occurrences, $\lambda$ is the frequency of occurrence, and $P\left( k \right)$ is the probability of $k$ occurrences. The Poisson distribution guides single-cell encapsulation of cells or particles in droplets: by diluting the input culture such that on average 1 encapsulation occurs in every 10 droplets ($\lambda$ = 0.1), the probability that multiple cells are encapsulated is limited ($P\left( k\geq2 \right)<0.05$). The Poisson frequency is converted to a bulk cell concentration by using characteristics of the droplet generator:

$$[X]=60 \frac{\omega\lambda}{Q_{d}}$$

where $[X]$ is the concentration of cells $\left( \frac{cells}{\mu L} \right)$, $\lambda$ is the Poisson frequency $\left( \frac{cells}{droplets} \right)$, $\omega$ is the droplet generation rate $\left( \frac{droplets}{s} \right)$, and $Q_{d}$ is the flow rate $\left( \frac{\mu L}{min} \right)$ for the droplet media.

In more complex assays where multiple groups are co-encapsulated at the single cell level, the Poisson distribution severely limits the sample throughput. For example, if a user wanted two different cell types co-encapsulated within a droplet, if the frequency of each was again set to 1 cell per 10 droplets ($\lambda$ = 0.1), only 1% of droplets would contain both cell types. This occurrence decreases by a factor of 10 as the number of unique single particles per droplet increases. Methods exist that encapsulate particles or cells at a higher rate than a Poisson process via inertial focusing or active droplet generation with lasers, however, these methods add operational or infrastructure requirements that limit its broad application^1–4^. Therefore, Poisson-governed encapsulation of single cells within droplets remains the most common method to study single cells in droplet microfluidics.

**Supplementary Note 2: Software**

Installation instructions:

1. Install python3.
2. Install pip with the following command prompt.

curl https://bootstrap.pypa.io/get-pip.py -o get-pip.py

python get-pip.py

1. Clone this repository.

git clone https://github.com/CIDARLAB/drop2image.git

cd drop2image

1. Install required libraries and run the program.

pip install -r requirements.txt

How to use Arduino script:

1. Open Serial_Arduino.ino in Arduino IDE.
2. Choose Board and Port that corresponds to your Arduino/ESP under Tools. Shown in Supplemental Figure 4A
3. Upload the code to the board using the arrow button.

How to use python version (pixel_art_gui.py):

1. Install required libraries and run the program. The GUI is shown in Supplemental Figure 4B.
   - cd drop2image
   - python3 pixel_art_gui.py
2. To load an image, click on load. When load is selected, a pop-up window to select the image will show up. Choose an image.
   - The image is resized to the user-specified mosaic size and rendered into a large-scale pixel array using the closest droplet color. The closest color is defined by high and low levels of red, green, and blue.
   - Images with *.png format are accepted for upload.
3. To draw or edit an image, choose a color and click on individual pixels. The default color is gray. If you color a pixel as skip, that pixel will be ignored when saved.
4. To save an image, click on save.
   - Image will save to current directory.
   - If you click save as, you can choose where to save.
5. To partition image into 5x5 panels for droplet generation, click on partition mosaic.
   - Saved partitioned images follow the convention filename_[row]_[column].png
   - The unique patterns are identified, labeled, and saved. Unique patterns are defined as panels that have different colors for at least one of the 25 pixels.
6. To send color information to Arduino, choose the device in drop-down and click send.
   - To send a single panel to the Arduino, the pixel array size in the software is set to 5x5 and the panel is uploaded and sent to the Arduino.
   - For the Arduino communication, the **Serial_Arduino.ino** is uploaded to the Arduino Uno, and the Serial Monitor is opened.
   - If you are a Windows user, device usually starts with COM.
   - If you are a Mac user, device usually starts with /dev/cu.
   - This will send the index of color in the color set.
   - To make sure that send is working, click on Serial Monitor at top-left corner. Serial Monitor buttom is circled in Supplemental Figure 4C.

How to use Local Webpage Version:

1. Install required libraries and run the program. The GUI is shown I n Supplemental Figure 4D.
   - cd drop2image
   - cd main
   - python3 main.py
2. To upload an image, click on upload image button and choose your image file. When load is selected, a pop-up window to select the image will show up. Choose an image. Image selection pop-up window is shown in Supplemental Figure 4E.
   - The image is resized to the user-specified mosaic size and rendered into a large-scale pixel array using the closest droplet color. The closest color is defined by high and low levels of red, green, and blue.
   - Images with *.png format are accepted for upload.
   - Loaded image is shown in Supplemental Figure 4F.
3. To draw or edit an image, choose a color and click on individual pixels. The default color is black. If you color a pixel as skip, that pixel will be ignored when saved. The default size of grid is 50x50.
4. To save an image, click on save image. Image will save to current directory.

**Supplemental Note 3: Methods for Screening of Protein Engineering and Genetic Circuits**

The following steps describe the process for protein engineering screening using a fluorescence output. The microfluidic chips used for this process are shown in Supplemental Figure 11A. The following materials, equipment and reagents are needed for this experiment:

| Item | Quantity |
| --- | --- |
| NORM JECT Luer Lock 2 mL Syringes | 7 |
| BioRad Droplet Generation Oil | 6 mL |
| Media fluid | 6 mL |
| Tygon NP 100-80 Microbore Tubing (ID: 0.03”) | 15 x 80 cm and 3 x 40 cm |
| Aquapel | 1 |
| HFE-7500 oil | 85 mL |
| 5 mL microcentrifuge test tube | 3 |
| 15 mL conical test tube | 5 |
| 50 mL conical test tube | 1 |
| Clear 96-well plate | 1 |
| Water bath | 1 |

| Equipment | Quantity |
| --- | --- |
| Harvard Apparatus Phd 2000 Syringe Pumps | 2 |
| Vortex | 1 |
| IDT X-Stream high speed camera | 1 |
| Computer | 1 |
| Zeiss Axiovert 200 M Inverted Microscope | 1 |
| BioTek Synergy H1 Microplate Reader | 1 |
| Upconverter circuit | 1 |
| Fluigent FlowEZ Pressure pumps | 2 |
| Fluigent LineUp Push-Pull Pressure pumps | 1 |
| Siglent SPD 3303X-E Programmable DC Power Supply | 1 |
| Nikon Eclipse Ti2-E Fluorescence Microscope | 1 |
| Thor Labs FT200EMT Optical Fibers | 4 |
| OptoEngine 50 mW lasers (405/488/561) | 3 |
| Thor Labs PMT | 1 |
| Semrock 446/523/600/677 bandpass filters | 1 |
| Arduino Uno Microcontroller | 1 |
| Incubator | 1 |

1. Transform mutagenesis library into cells (off-chip).
2. Encapsulate cells in droplets.
   1. Fill 2 syringes with BioRad Droplet Generation Oil and label syringes as “BioRad oil.” Load syringes into syringe pumps and enter syringe diameter. Connect tubing to syringes and oil ports on the droplet generation microfluidic device.
   2. Fill a syringe with media and label syringes as “Media.” Load syringe into syringe pumps and enter syringe diameter. Connect tubing to the syringe and the media fluid port on the droplet generation microfluidic device.
   3. Prepare a 5 mL test tube and one piece of tubing for droplet generation and storage by coating the inside with Aquapel. Remove excess aquapel by rinsing the test tube with HFE-7500 Oil and vortexing. Label test tube as “Droplets.”
   4. Label a 15 mL test tube as “Waste.” Connect the surface treated tubing to the outlet on the droplet generation microfluidic device and place the other end of the tubing in the waste test tube.
   5. Remove any air bubbles from the ports and channels. Set the oil flow rate to 10 ul/min and run for about 1 minute. Then, set the media flow rate to 10 ul/min and let that run for about 1 minute. If needed to remove additional air bubbles, set the flow rates to slightly higher rates.
   6. Set the flow rates to yield ~1 nL droplets with media and BioRad Droplet Generation Oil. Determine the droplet generation rate by taking a video of the droplets and counting the number of droplets generated in 1 second. Note the media flow rate in the spreadsheet. Calculate the droplet volume ($v$) and droplet diameter ($d$) from the droplet generation rate ($w$) and media volumetric flow rate ($Q_{d}$) with the following equations:
      $v= \frac{Q_{d}}{w}$
      $d= \sqrt[3]{\frac{6v}{\pi}}$
   7. Calculate the required cell concentration based on the droplet generation rate and the media flow rate with the following equation, where $[X]$ is the concentration of cells $\left( \frac{\mathrm{cells}}{\mu L} \right)$, $\lambda$ is the Poisson frequency $\left( \frac{\mathrm{cells}}{\mathrm{droplets}} \right)$, $\omega$ is the droplet generation rate $\left( \frac{\mathrm{droplets}}{s} \right)$, and $Q_{d}$ is the media flow rate $\left( \frac{\mu L}{\min} \right)$.
      $[X]=60 \frac{\omega\lambda}{Q_{d}}$
   8. Measure the Optical Density (OD) of the cell sample using a plate reader. Convert the OD to cell concentration using a conversion factor for E. coli $\left( 2.88 x {10}^{8}\frac{\mathrm{cells}}{mL*OD} \right)$ or S. cerevisiae $\left( 1 x {10}^{7}\frac{\mathrm{cells}}{mL*OD} \right)$ in the spreadsheet.
   9. Dilute the cell sample in media from the estimated cell concentration (from Optical Density measurement) to the calculated cell concentration for droplet generation.
   10. Fill a syringe with media and label syringes as “Media with Cells.” Load syringe into syringe pumps and enter syringe diameter. Connect tubing to the syringe and the media fluid port on the droplet generation microfluidic device.
   11. Set the flow rates as droplet generation flow rates in Step F.
   12. Once droplets are generated at a steady state and the appropriate size, disconnect the output tubing from the microfluidic device and empty the contents into waste test tube. Reconnect the output tubing to the microfluidic device and place other end of the tubing in the test tube labeled “Droplets” to collect droplets.
   13. Run droplet generation until enough droplets have been generated for downstream analysis.
   14. Carefully disconnect the output tubing and gently empty the remaining droplets into the test tube labeled “Droplets.”
3. Incubate droplets.
   1. Add a water bath to the incubator to saturate the humidity and prevent evaporation from the droplets.
   2. For E. coli or S. cerevisiae set the incubator to 37°C or 30°C, respectively. Preheat incubator for approximately 1 hour.
   3. Incubate droplets for 6 hours and note the incubation start/end time on the spreadsheet.
4. Picoinject fluorogenic substrate into droplets.
   1. Prepare a 5 mL test tube and 2 pieces of 80 cm tubing for droplet generation and storage by coating the inside with Aquapel. Remove excess aquapel by rinsing test tube with HFE-7500 Oil and vortexing. Label test tube as “Picoinjected Droplets.” Label a 15 mL additional test tube as “Waste.” Connect one of the surface treated pieces of tubing to the picoinjection microfluidic device outlet and place other end of tubing in test tube labeled “Waste.”
   2. Fill a 50 mL test tube with HFE 7500, label test tube as “HFE oil,” and tap 3 ports into the test tube top. Connect one piece of 40 cm of tubing from the pressure pump outlet to a port on the test tube top, ensuring the end of the tubing is placed above the oil. Connect 2 pieces of 80 cm of tubing to the test tube top, ensuring the end of the tubing is at the bottom of the test tube.
   3. Fill a 15 mL test tube with HFE 7500, label test tube as “Oil for Reinjected Droplets,” and tap 2 ports into the test tube top. Connect one piece of 40 cm of tubing from the pressure pump outlet to a port on the test tube top, ensuring the end of the tubing is above the oil. Connect the one of the surface treated tubing to the test tube top, ensuring the end of the tubing is at the bottom of the test tube.
   4. Fill a 15 mL test tube with the fluorogenic substrate, label test tube as “Fluorogenic Substrate,” and tap 2 ports into the test tube top. Connect one piece of 40 cm of tubing from the pressure pump outlet to a port on the test tube top, ensuring the end of the tubing is placed above the fluid. Connect one piece of 80 cm tubing to the test tube top, ensuring the end of the tubing is at the bottom of the test tube.
   5. Connect voltage upconverter to the picoinjection microfluidic device.
   6. Set the oil pressure pump (connected to the “HFE oil” test tube) to a positive pressure to start the oil flowing through the tubing. Once the oil has reached the end of the tubing, connect the tubing to the oil ports on the picoinjection microfluidic chip. Flow oil through picoinjection chip to remove any air bubbles trapped in the channels or ports.
   7. Set the fluorogenic substrate pressure pump (connected to the “fluorogenic substrate” test tube) to a positive pressure to start the fluid flowing through the tubing. Once the fluid has reached the end of the tubing, connect the tubing to the injection port on the picoinjection microfluidic chip.
   8. Set the pressure pump (connected to the “Oil for Reinjected Droplets” test tube) to a positive pressure to start the oil flowing through the surface treated tubing. Once the oil is 2 cm from the end of the tubing, set the pressure pump to a slight negative pressure and aspirate the incubated droplets, leaving a small air bubble between oil and droplets. Collect about 1 mL of droplets into the tubing, while monitoring the first droplets collected to ensure droplets have not reached the test tube. Once the droplets are in the tubing, connect the tubing to the droplet input port on the picoinjection microfluidic chip to reinject the droplets.
   9. Slowly increase the pressure of the HFE oil and the reinjected droplets, until droplets are reinjected at a steady state of approximately 100 Hz. While monitoring the droplet reinjection, slowly increase the pressure of the fluorogenic substrate to ensure droplets or oil are not going through to the injection port. Droplets and oil should be flowing out through the microfluidic device outlet.
   10. Once droplets are reinjected into microfluidic device at a steady state, turn voltage upconverter on and slowly increase the pressure of the fluorogenic substrate. Observe the increase in diameter of droplets as the pressure increases.
   11. Once the diameter has increased by 25%, disconnect the output tubing from the microfluidic device and empty the contents into “Waste” test tube. Reconnect the output tubing to the microfluidic device and place other end of the tubing in the test tube labeled “Picoinjected Droplets” to collect droplets. Note the pressures and reinjection rate in the spreadsheet.
   12. Continue monitoring the droplet reinjection and droplet volume change until about 1 mL of droplets have been picoinjected.
   13. Carefully disconnect the output tubing and gently empty the remaining droplets into the test tube labeled “Picoinjected Droplets.”
5. Incubate droplet.
   1. Add a water bath to the incubator to saturate the humidity and prevent evaporation from the droplets.
   2. For E. coli or S. cerevisiae set the incubator to 37°C or 30°C, respectively. Preheat incubator for approximately 1 hour.
   3. Incubate droplets for 12 hours and note the incubation start/end time on the spreadsheet.
6. Reinject droplets into single-channel fluorescence-activated droplet sorting device.
   1. Prepare a 5 mL test tube and one piece of tubing for droplet storage by coating the inside with Aquapel. Remove excess aquapel by rinsing test tube with HFE-7500 Oil and vortexing. Label test tube as “Sorted Droplets.”
   2. Fill 2 syringes with HFE 7500 and label syringes as “HFE oil.” Load syringe into syringe pumps and enter syringe diameter. Connect tubing to syringes and oil ports on the fluorescence-activated droplet sorting microfluidic device.
   3. Fill a syringe with 2 mL of HFE 7500 and label syringes as “Droplets.” Connect the surface treated tubing to syringe. Load syringes into syringe pumps and enter syringe diameter.
   4. Insert optical fibers into the fluorescence-activated droplet sorting microfluidic device for the laser and PMT. Use the laser and bandpass filters that correspond to the fluorescent output of the biological constructs.
   5. Connect voltage upconverter circuit to the fluorescence-activated droplet sorting microfluidic device and Arduino.
   6. Set the syringe pump (with the syringe labeled “Droplets”) to refill at 100 uL/min. Once the oil is 2 cm from the end of the tubing, aspirate the incubated droplets leaving a small air bubble between the oil and the droplets. Collect about 1 mL of droplets into tubing, while monitoring the first droplets collected to ensure droplets have not reached the end of the tubing connected to the syringe. Once the droplets are in the tubing, connect the tubing to the droplet input port on the fluorescence-activated droplet sorting microfluidic chip to reinject the droplets.
   7. Set syringe pumps to reinject droplets at a periodic rate by first flowing the oil to flush out any air bubbles and then start flowing the droplets.
   8. Once droplets are reinjected into the fluorescence-activated droplet sorting microfluidic device at a steady state, adjust the laser intensity until the droplets are detected by the PMT. Adjust the sorting threshold to positively sort the most fluorescent droplets. Adjust the delay between fluorescence data processing and sorting actuation to correctly sort fluorescent droplets. Adjust upconverter voltage as needed depending on droplet flow rate. Note the flow rates, laser intensity, sorting threshold, fluorescence data processing and sorting delay, and upconverter voltage in spreadsheet.
7. Sequence positively sorted droplets (off-chip).

The following steps describe the process for genetic circuit evaluation screening using a fluorescence output. This process was adapted from Holtstein et. al. cell-free directed evolution screening^6^. The microfluidic chips used for this process are shown in Supplemental Figure 11B. The following materials, equipment and reagents are needed for this experiment:

| Item | Quantity |
| --- | --- |
| NORM JECT Luer Lock 2 mL Syringes | 7 |
| BioRad Droplet Generation Oil | 6 mL |
| Media fluid | 6 mL |
| Tygon NP 100-80 Microbore Tubing (ID: 0.03”) | 20 x 80 cm and 6 x 40 cm |
| Aquapel | 1 |
| HFE-7500 oil | 155 mL |
| 5 mL microcentrifuge test tube | 4 |
| 15 mL conical test tube | 8 |
| 50 mL conical test tube | 2 |
| Water bath | 1 |

| Equipment | Quantity |
| --- | --- |
| Harvard Apparatus Phd 2000 Syringe Pumps | 2 |
| Vortex | 1 |
| IDT X-Stream high speed camera | 1 |
| Computer | 1 |
| Zeiss Axiovert 200 M Inverted Microscope | 1 |
| BioTek Synergy H1 Microplate Reader | 1 |
| Upconverter circuit | 1 |
| Fluigent FlowEZ Pressure pumps | 2 |
| Fluigent LineUp Push-Pull Pressure pumps | 1 |
| Siglent SPD 3303X-E Programmable DC Power Supply | 1 |
| Nikon Eclipse Ti2-E Fluorescence Microscope | 1 |
| Thor Labs FT200EMT Optical Fibers | 4 |
| OptoEngine 50 mW lasers (405/488/561) | 3 |
| Thor Labs PMT | 1 |
| Semrock 446/523/600/677 bandpass filters | 1 |
| Arduino Uno Microcontroller | 1 |
| Incubator | 1 |

1. Prepare plasmids for co-encapsulation (off-chip).
2. Co-encapsulate plasmids and rolling circle amplification mix using a two-input droplet generator. Set flow rates to yield 1 nL droplets.
   1. Fill 2 syringes with BioRad Droplet Generation Oil and label syringes as “BioRad oil.” Load syringes into syringe pumps and enter syringe diameter. Connect tubing to syringes and oil ports on the droplet generation microfluidic device.
   2. Fill a syringe with cell-free mix and label syringes as “cell-free mix.” Load syringe into syringe pumps and enter syringe diameter. Connect tubing to the syringe and the cell-free mix fluid port on the droplet generation microfluidic device.
   3. Prepare a 5 mL test tube and one piece of tubing for droplet generation and storage by coating the inside with Aquapel. Remove excess aquapel by rinsing the test tube with HFE-7500 Oil and vortexing. Label test tube as “Droplets.”
   4. Label a 15 mL test tube as “Waste.” Connect the surface treated tubing to the outlet on the droplet generation microfluidic device and place the other end of the tubing in the waste test tube.
   5. Remove any air bubbles from the ports and channels. Set the oil flow rate to 10 ul/min and run for about 1 minute. Then, set the cell-free mix flow rate to 10 ul/min and let that run for about 1 minute. If needed to remove additional air bubbles, set the flow rates to slightly higher rates.
   6. Set the flow rates to yield ~1 nL droplets with cell-free mix and BioRad Droplet Generation Oil. Determine the droplet generation rate by taking a video of the droplets and counting the number of droplets generated in 1 second. Note the cell-free mix flow rate. Calculate the droplet volume ($v$) and droplet diameter ($d$) from the droplet generation rate ($w$) and cell-free mix volumetric flow rate ($Q_{d}$) with the following equations:
      $v= \frac{Q_{d}}{w}$
      $d= \sqrt[3]{\frac{6v}{\pi}}$
   7. Calculate the required cell concentration based on the droplet generation rate and the cell-free mix flow rate with the following equation, where $[X]$ is the concentration of cells $\left( \frac{\mathrm{cells}}{\mu L} \right)$, $\lambda$ is the Poisson frequency $\left( \frac{\mathrm{cells}}{\mathrm{droplets}} \right)$, $\omega$ is the droplet generation rate $\left( \frac{\mathrm{droplets}}{s} \right)$, and $Q_{d}$ is the cell-free mix flow rate $\left( \frac{\mu L}{\min} \right)$.
      $[X]=60 \frac{\omega\lambda}{Q_{d}}$
   8. Dilute the plasmids in cell-free mix to the calculated plasmid concentration for droplet generation.
   9. Fill a syringe with cell-free mix and label syringes as “cell-free mix with plasmid.” Load syringe into syringe pumps and enter syringe diameter. Connect tubing to the syringe and the cell-free mix fluid port on the droplet generation microfluidic device.
   10. Set the flow rates as droplet generation flow rates in Step F.
   11. Once droplets are generated at a steady state and the appropriate size, disconnect the output tubing from the microfluidic device and empty the contents into waste test tube. Reconnect the output tubing to the microfluidic device and place other end of the tubing in the test tube labeled “Droplets” to collect droplets.
   12. Run droplet generation until enough droplets have been generated for downstream analysis.
   13. Carefully disconnect the output tubing and gently empty the remaining droplets into the test tube labeled “Droplets.”
3. Incubate droplets.
   1. Add a water bath to the incubator to saturate the humidity and prevent evaporation from the droplets.
   2. For E. coli or S. cerevisiae set the incubator to 37°C or 30°C, respectively. Preheat incubator for approximately 1 hour.
   3. Incubate droplets for 6 hours and note the incubation start/end time on the spreadsheet.
4. Picoinject in vitro transcription and translation reagent into droplets. Set droplet reinjection and picoinjection pressures to double the droplet volume.
   1. Prepare a 5 mL test tube and 2 pieces of 80 cm tubing for droplet generation and storage by coating the inside with Aquapel. Remove excess aquapel by rinsing test tube with HFE-7500 Oil and vortexing. Label test tube as “Picoinjected Droplets.” Label a 15 mL additional test tube as “Waste.” Connect one of the surface treated pieces of tubing to the picoinjection microfluidic device outlet and place other end of tubing in test tube labeled “Waste.”
   2. Fill a 50 mL test tube with HFE 7500, label test tube as “HFE oil,” and tap 3 ports into the test tube top. Connect one piece of 40 cm of tubing from the pressure pump outlet to a port on the test tube top, ensuring the end of the tubing is placed above the oil. Connect 2 pieces of 80 cm of tubing to the test tube top, ensuring the end of the tubing is at the bottom of the test tube.
   3. Fill a 15 mL test tube with HFE 7500, label test tube as “Oil for Reinjected Droplets,” and tap 2 ports into the test tube top. Connect one piece of 40 cm of tubing from the pressure pump outlet to a port on the test tube top, ensuring the end of the tubing is above the oil. Connect the one of the surface treated tubing to the test tube top, ensuring the end of the tubing is at the bottom of the test tube.
   4. Fill a 15 mL test tube with the in vitro transcription and translation reagent, label test tube as “in vitro transcription and translation reagent,” and tap 2 ports into the test tube top. Connect one piece of 40 cm of tubing from the pressure pump outlet to a port on the test tube top, ensuring the end of the tubing is placed above the fluid. Connect one piece of 80 cm tubing to the test tube top, ensuring the end of the tubing is at the bottom of the test tube.
   5. Connect voltage upconverter to the picoinjection microfluidic device.
   6. Set the oil pressure pump (connected to the “HFE oil” test tube) to a positive pressure to start the oil flowing through the tubing. Once the oil has reached the end of the tubing, connect the tubing to the oil ports on the picoinjection microfluidic chip. Flow oil through picoinjection chip to remove any air bubbles trapped in the channels or ports.
   7. Set the in vitro transcription and translation reagent pressure pump (connected to the “in vitro transcription and translation reagent” test tube) to a positive pressure to start the fluid flowing through the tubing. Once the fluid has reached the end of the tubing, connect the tubing to the injection port on the picoinjection microfluidic chip.
   8. Set the pressure pump (connected to the “Oil for Reinjected Droplets” test tube) to a positive pressure to start the oil flowing through the surface treated tubing. Once the oil is 2 cm from the end of the tubing, set the pressure pump to a slight negative pressure and aspirate the incubated droplets, leaving a small air bubble between oil and droplets. Collect about 1 mL of droplets into the tubing, while monitoring the first droplets collected to ensure droplets have not reached the test tube. Once the droplets are in the tubing, connect the tubing to the droplet input port on the picoinjection microfluidic chip to reinject the droplets.
   9. Slowly increase the pressure of the HFE oil and the reinjected droplets, until droplets are reinjected at a steady state of approximately 100 Hz. While monitoring the droplet reinjection, slowly increase the pressure of the in vitro transcription and translation reagent to ensure droplets or oil are not going through to the injection port. Droplets and oil should be flowing out through the microfluidic device outlet.
   10. Once droplets are reinjected into microfluidic device at a steady state, turn voltage upconverter on and slowly increase the pressure of the in vitro transcription and translation reagent. Observe the increase in diameter of droplets as the pressure increases.
   11. Once the diameter has increased by 25%, disconnect the output tubing from the microfluidic device and empty the contents into “Waste” test tube. Reconnect the output tubing to the microfluidic device and place other end of the tubing in the test tube labeled “Picoinjected Droplets” to collect droplets. Note the pressures and reinjection rate in the spreadsheet.
   12. Continue monitoring the droplet reinjection and droplet volume change until about 1 mL of droplets have been picoinjected.
   13. Carefully disconnect the output tubing and gently empty the remaining droplets into the test tube labeled “Picoinjected Droplets.”
5. Incubate droplets.
   1. Add a water bath to the incubator to saturate the humidity and prevent evaporation from the droplets.
   2. For E. coli or S. cerevisiae set the incubator to 37°C or 30°C, respectively. Preheat incubator for approximately 1 hour.
   3. Incubate droplets for 12 hours and note the incubation start/end time on the spreadsheet.
6. Picoinject fluorogenic substrate into droplets. Set droplet reinjection and picoinjection pressures to double the droplet volume.
   1. Prepare a 5 mL test tube and 2 pieces of 80 cm tubing for droplet generation and storage by coating the inside with Aquapel. Remove excess aquapel by rinsing test tube with HFE-7500 Oil and vortexing. Label test tube as “Picoinjected Droplets.” Label a 15 mL additional test tube as “Waste.” Connect one of the surface treated pieces of tubing to the picoinjection microfluidic device outlet and place other end of tubing in test tube labeled “Waste.”
   2. Fill a 50 mL test tube with HFE 7500, label test tube as “HFE oil,” and tap 3 ports into the test tube top. Connect one piece of 40 cm of tubing from the pressure pump outlet to a port on the test tube top, ensuring the end of the tubing is placed above the oil. Connect 2 pieces of 80 cm of tubing to the test tube top, ensuring the end of the tubing is at the bottom of the test tube.
   3. Fill a 15 mL test tube with HFE 7500, label test tube as “Oil for Reinjected Droplets,” and tap 2 ports into the test tube top. Connect one piece of 40 cm of tubing from the pressure pump outlet to a port on the test tube top, ensuring the end of the tubing is above the oil. Connect the one of the surface treated tubing to the test tube top, ensuring the end of the tubing is at the bottom of the test tube.
   4. Fill a 15 mL test tube with the fluorogenic substrate, label test tube as “fluorogenic substrate,” and tap 2 ports into the test tube top. Connect one piece of 40 cm of tubing from the pressure pump outlet to a port on the test tube top, ensuring the end of the tubing is placed above the fluid. Connect one piece of 80 cm tubing to the test tube top, ensuring the end of the tubing is at the bottom of the test tube.
   5. Connect voltage upconverter to the picoinjection microfluidic device.
   6. Set the oil pressure pump (connected to the “HFE oil” test tube) to a positive pressure to start the oil flowing through the tubing. Once the oil has reached the end of the tubing, connect the tubing to the oil ports on the picoinjection microfluidic chip. Flow oil through picoinjection chip to remove any air bubbles trapped in the channels or ports.
   7. Set the fluorogenic substrate pressure pump (connected to the “fluorogenic substrate” test tube) to a positive pressure to start the fluid flowing through the tubing. Once the fluid has reached the end of the tubing, connect the tubing to the injection port on the picoinjection microfluidic chip.
   8. Set the pressure pump (connected to the “Oil for Reinjected Droplets” test tube) to a positive pressure to start the oil flowing through the surface treated tubing. Once the oil is 2 cm from the end of the tubing, set the pressure pump to a slight negative pressure and aspirate the incubated droplets, leaving a small air bubble between oil and droplets. Collect about 1 mL of droplets into the tubing, while monitoring the first droplets collected to ensure droplets have not reached the test tube. Once the droplets are in the tubing, connect the tubing to the droplet input port on the picoinjection microfluidic chip to reinject the droplets.
   9. Slowly increase the pressure of the HFE oil and the reinjected droplets, until droplets are reinjected at a steady state of approximately 100 Hz. While monitoring the droplet reinjection, slowly increase the pressure of the fluorogenic substrate to ensure droplets or oil are not going through to the injection port. Droplets and oil should be flowing out through the microfluidic device outlet.
   10. Once droplets are reinjected into microfluidic device at a steady state, turn voltage upconverter on and slowly increase the pressure of the fluorogenic substrate. Observe the increase in diameter of droplets as the pressure increases.
   11. Once the diameter has increased by 25%, disconnect the output tubing from the microfluidic device and empty the contents into “Waste” test tube. Reconnect the output tubing to the microfluidic device and place other end of the tubing in the test tube labeled “Picoinjected Droplets” to collect droplets. Note the pressures and reinjection rate in the spreadsheet.
   12. Continue monitoring the droplet reinjection and droplet volume change until about 1 mL of droplets have been picoinjected.
   13. Carefully disconnect the output tubing and gently empty the remaining droplets into the test tube labeled “Picoinjected Droplets.”
7. Incubate droplets.
   1. Add a water bath to the incubator to saturate the humidity and prevent evaporation from the droplets.
   2. For E. coli or S. cerevisiae set the incubator to 37°C or 30°C, respectively. Preheat incubator for approximately 1 hour.
   3. Incubate droplets for 4 hours and note the incubation start/end time on the spreadsheet.
8. Reinject sorted droplets into single-channel fluorescence-activated droplet sorting and anchor grid.
   1. Prepare a 5 mL test tube and one piece of tubing for droplet storage by coating the inside with Aquapel. Remove excess aquapel by rinsing test tube with HFE-7500 Oil and vortexing. Label test tube as “Sorted Droplets.”
   2. Fill 2 syringes with HFE 7500 and label syringes as “HFE oil.” Load syringe into syringe pumps and enter syringe diameter. Connect tubing to syringes and oil ports on the anchoring microfluidic device.
   3. Fill a syringe with 2 mL of HFE 7500 and label syringes as “Droplets.” Connect the surface treated tubing to syringe. Load syringes into syringe pumps and enter syringe diameter.
   4. Insert optical fibers into the fluorescence-activated droplet sorting microfluidic device for the laser and PMT. Use the laser and bandpass filters that correspond to the fluorescent output of the biological constructs.
   5. Connect voltage upconverter circuit to the anchoring microfluidic device and Arduino.
   6. Set the syringe pump (with the syringe labeled “Droplets”) to refill at 100 uL/min. Once the oil is 2 cm from the end of the tubing, aspirate the incubated droplets leaving a small air bubble between the oil and droplets. Collect about 1 mL of droplets into tubing, while monitoring the first droplets collected to ensure droplets have not reached the end of the tubing connect to the syringe. Once the droplets are in the tubing, connect the tubing to the droplet input port on the anchoring microfluidic chip to reinject the droplets.
   7. Set syringe pumps to reinject droplets at a periodic rate by first flowing the oil to flush out any air bubbles and then start flowing the droplets.
   8. Once droplets are reinjected into anchoring microfluidic device at a steady state, adjust the laser intensity until the droplets are detected by the PMT. Adjust the sorting threshold to positively sort the most fluorescent droplets. Adjust the delay between fluorescence data processing and sorting actuation to correctly sort the fluorescence droplets into the anchoring channel.
   9. As droplet are positively sorted into the anchoring channel, ensure droplets are trapped in first anchor and knocked down to the next anchor as another droplet is sorted into anchoring channel. Note the flow rates, laser intensity, sorting threshold, fluorescence data processing and sorting delay, and upconverter voltage in spreadsheet.
   10. Observe fluorescence levels with scope to evaluate temporal dynamics of genetic circuit.


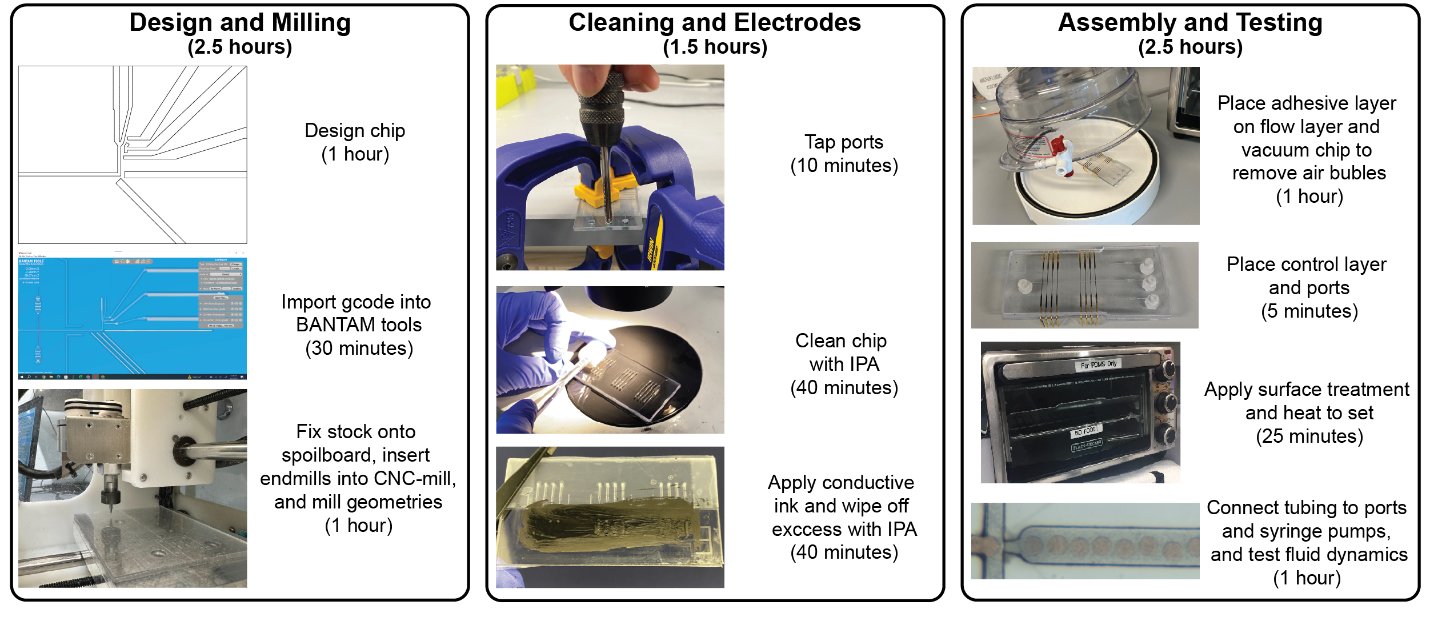


**Supplemental Figure 1: Rapid Prototyping Workflow.** First, the device is designed using Autodesk Fusion 360. Next, the device is milled using a Bantam Tools Desktop CNC-milling machine, followed by cleaning and placing conductive ink electrodes. Lastly, the devices are assembled and tested. The entire workflow can be completed within a day. See^5^ for an overview of desktop micromilling process.


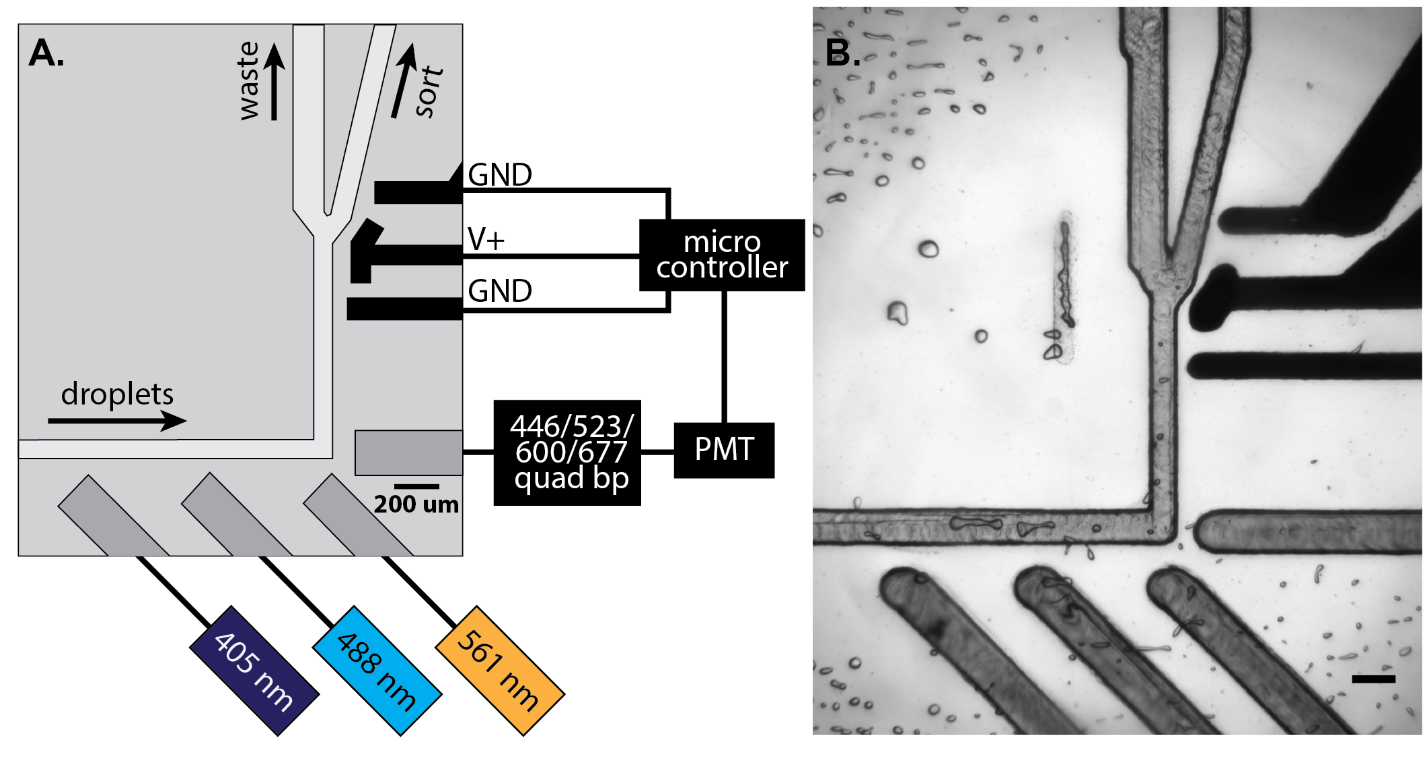


**Supplemental Figure 2: Overview of 3-channel fluorescent detection connected to a droplet sorter.** (A-B) Three excitation fibers are inserted into the device and collected with a single emission fiber inserted at a channel right angle turn to detect excitation from each laser. Stray light is filtered out through two quad bandpass filters and quantified by a photomultiplier tube. This signal is sent to a programmed microcontroller to actuate the sorting of droplets with target properties. Scale bars are 200 μm.


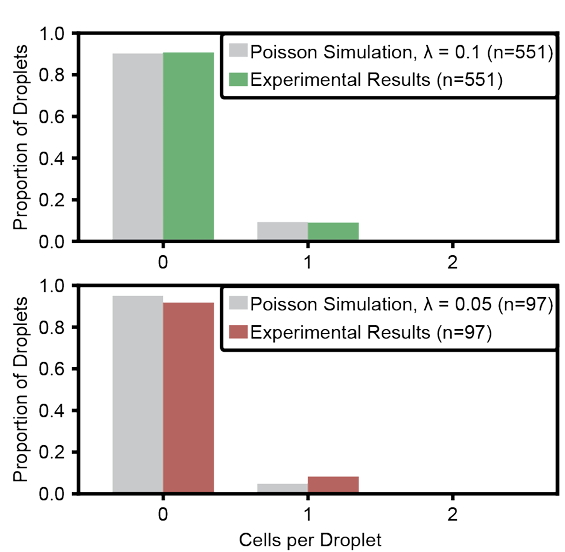


**Supplemental Figure 3: Cell encapsulation follows a Poisson Distribution.** Using the Poisson distribution, single cells of *E. coli* and *S. cerevisiae* are encapsulated within a microfluidic device. The number of *E. coli* (top) and *S. cerevisiae* (bottom) cells per droplet align with the estimates from the Possion distribution with a frequency of 0.1 and 0.05 cells/droplet, respectively, resulting in predicable cell encapsulation.


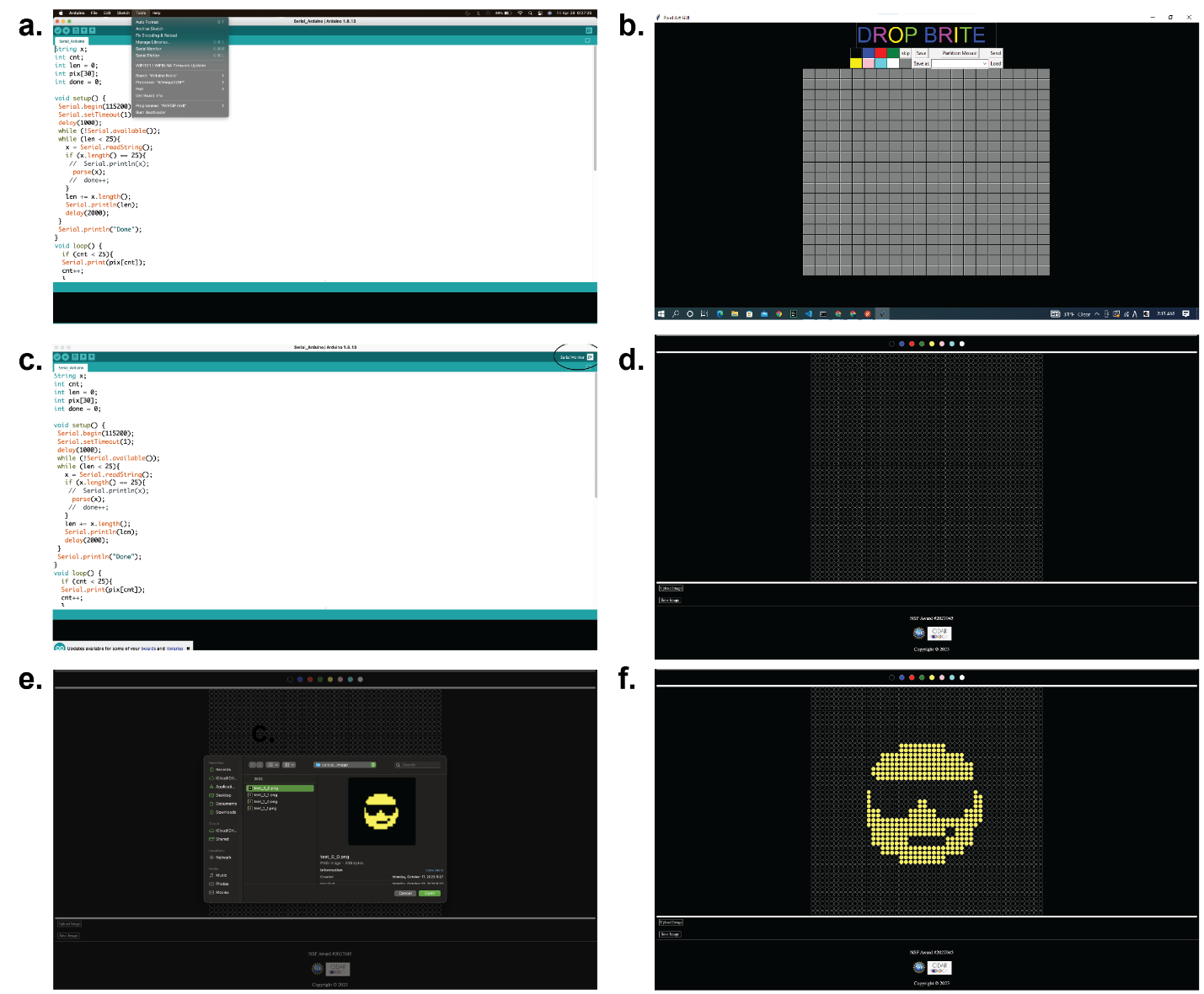


**Supplemental Figure 4: Custom image to pixel software.** (A) In order to send panels to Arduino, choose board and port that corresponds to Arduino/ESP under Tools and upload Arduino code to board using arrow button. (B) Drop Brite GUI from pixel_art_gui.py. To load an image, click on the load button. The image is resized to the user-specified mosaic size and rendered into a large-scale pixel array using the closest droplet color. The closest color is defined by high and low levels of red, green, and blue.(C) To send color information to Arduino, choose the device in drop-down and click send. To send a single panel to the Arduino, the pixel array size in the software is set to 5x5 and the panel is uploaded and sent to the Arduino. (D) Drop Brite GUI for local webpage version. (E) To upload an image, click on upload image button and choose your image file. When load is selected, a pop-up window to select the image will show up. Choose an image.(F) To draw or edit an image, choose a color and click on individual pixels. The default color is gray. If you color a pixel as skip, that pixel will be ignored when saved. Default color is black. Default size of grid is 50x50.


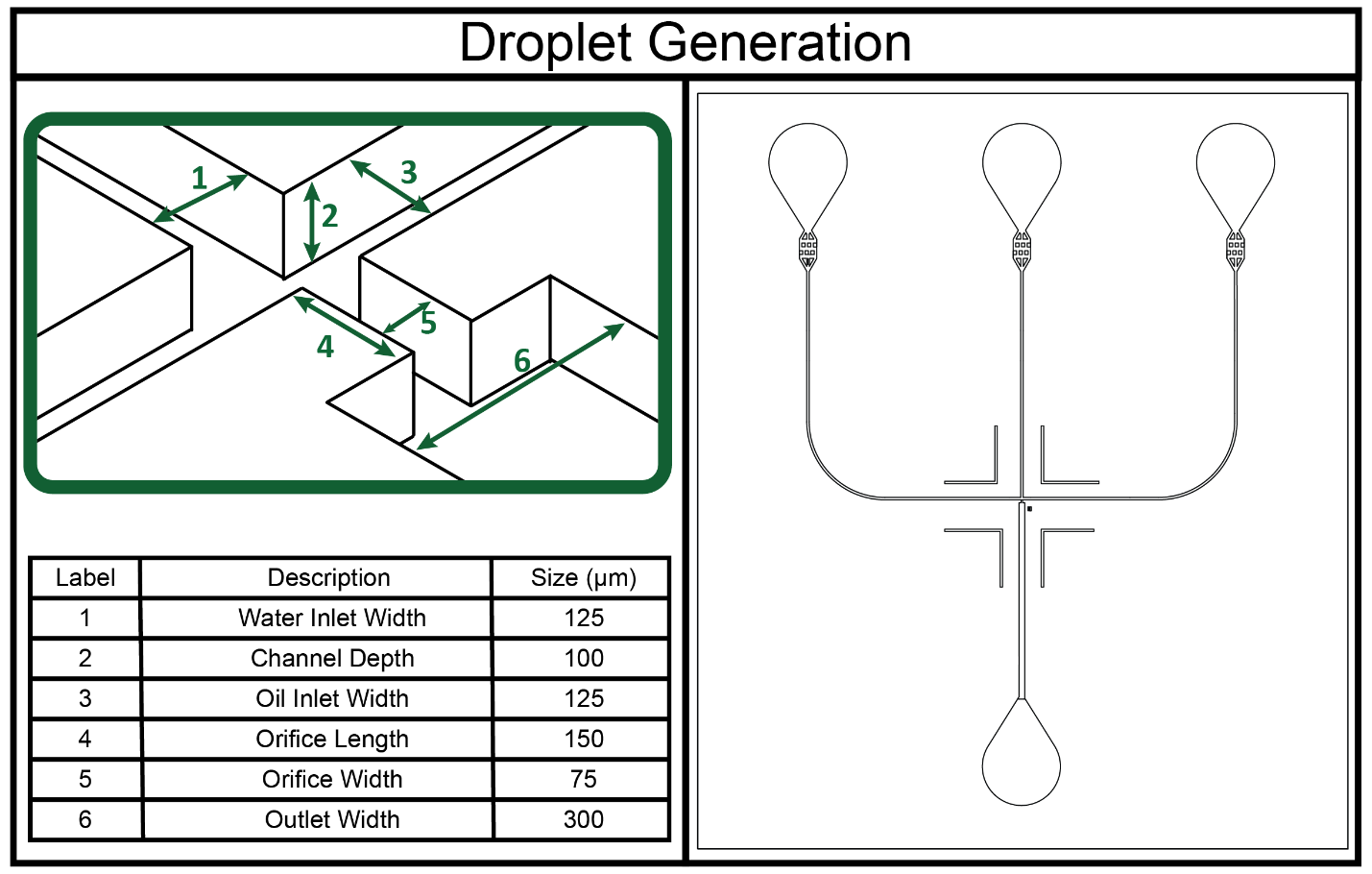


**Supplemental Figure 5: Data sheet for droplet generation.** Fusion 360 Link: <https://a360.co/3ZbmQry>

**
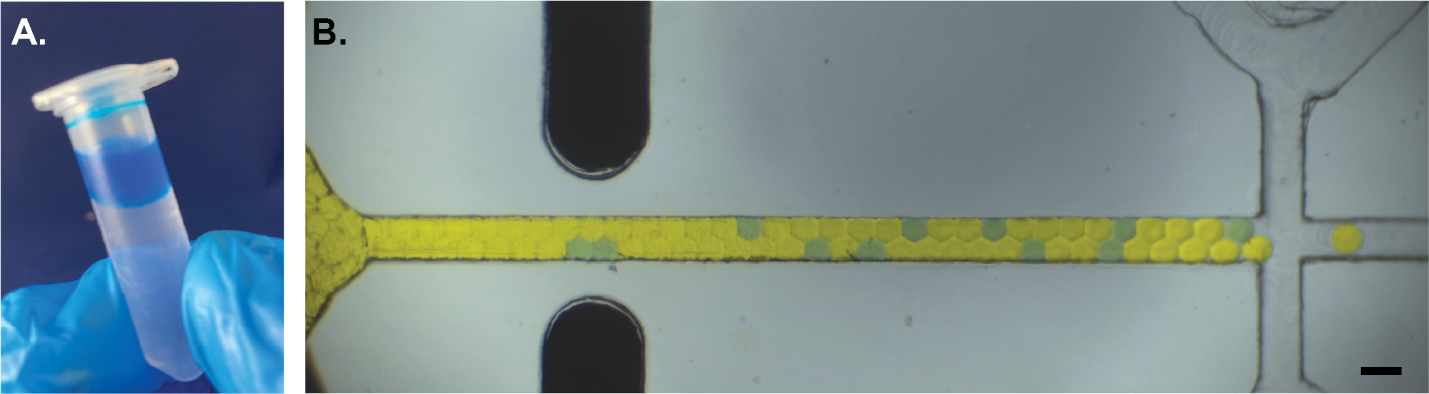
**

**Supplemental Figure 6: Overview of droplet storage and reinjection.** (A) After droplet generation, droplets can be immediately collected into a 2-mL test tube for incubation. These collected droplets are stable for multiple days (> 3 days) when stored within a high-humidity incubator to facilitate the growth of encapsulated cells or reaction progress. (B) Incubated droplets can then be stably reinjected into other microfluidic devices for further processing such as fluorescence sensing and sorting. The scale bar is 150 µm.


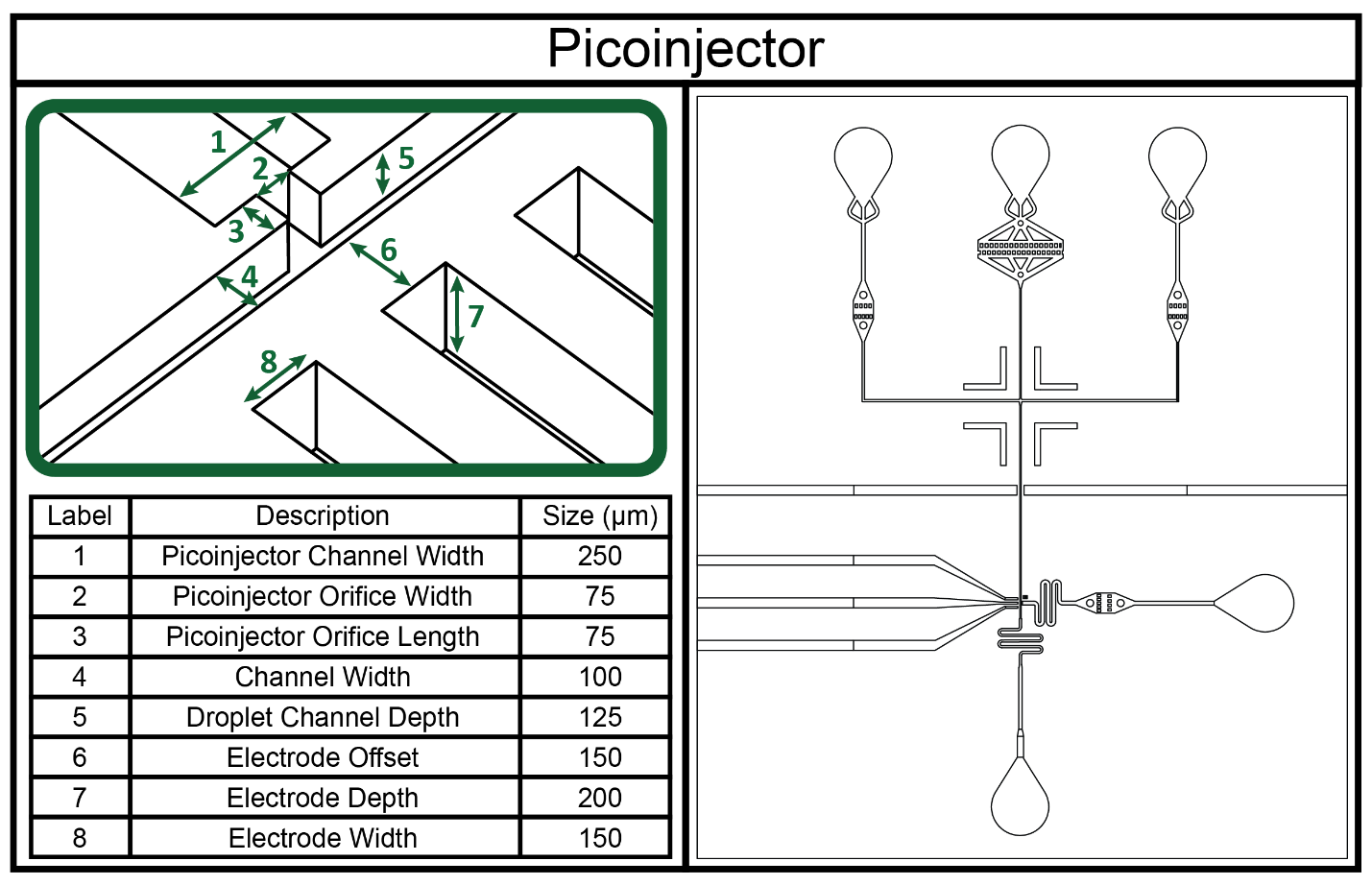


**Supplemental Figure 7: Data sheet for picoinjector.** Fusion 360 Link: <https://a360.co/3PaHoMp>


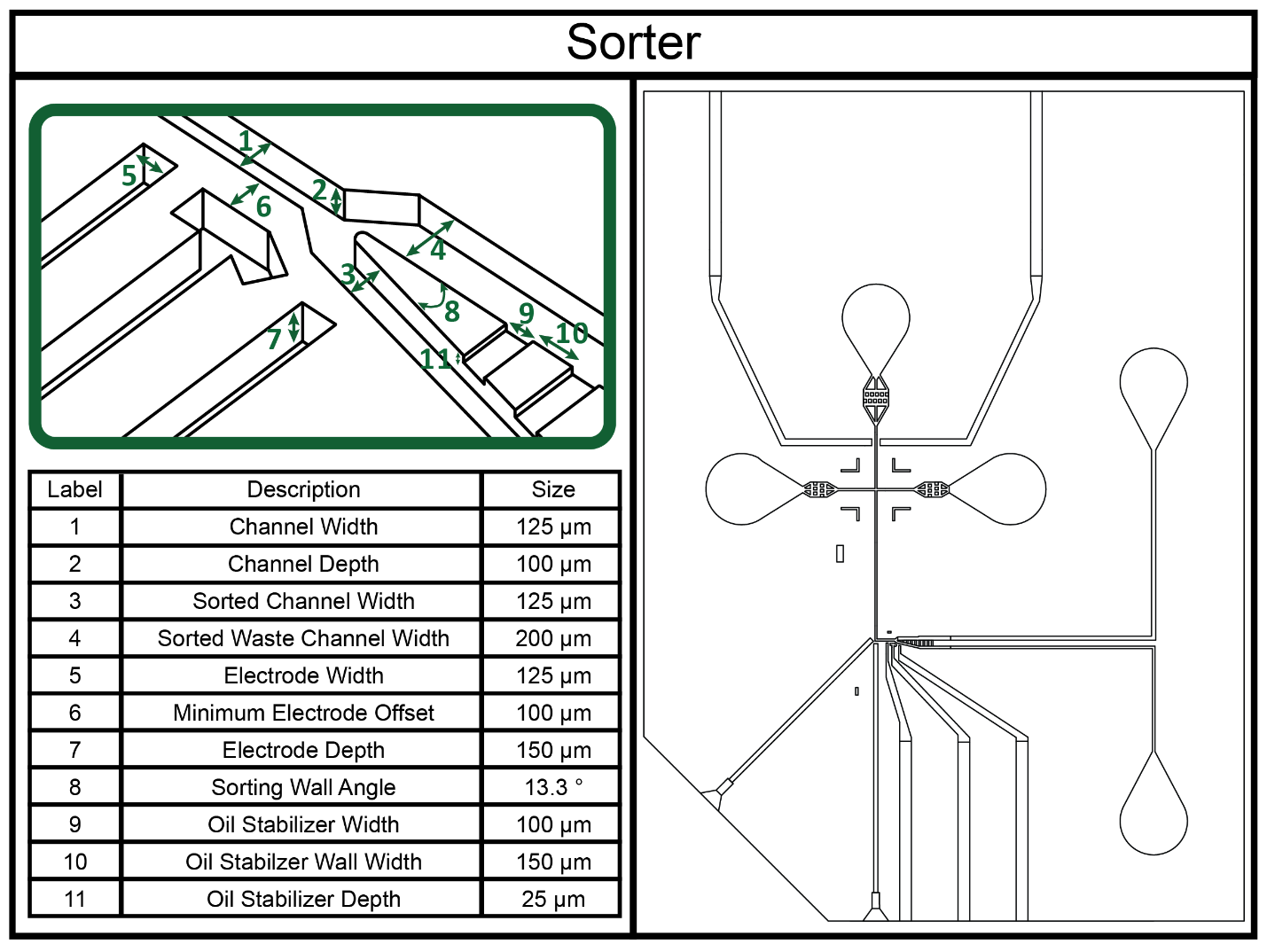


**Supplemental Figure 8:** Data sheet for sorting. Fusion 360 Link: <https://a360.co/3PIZJln>, <https://a360.co/48dy9nt>


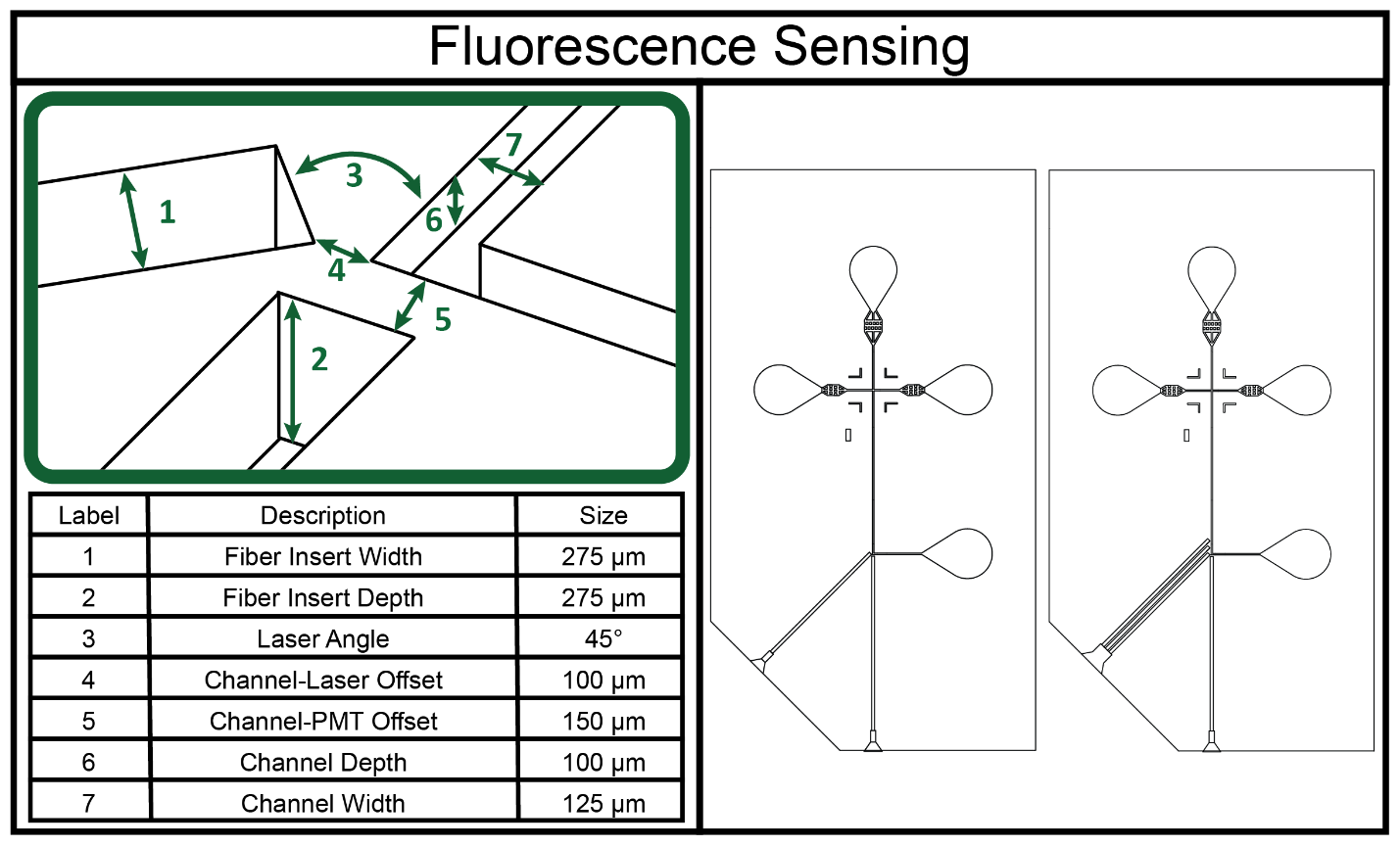


**Supplemental Figure 9: Data sheet for fluorescence sensing.** Fusion 360 Link: <https://a360.co/3PAjnzV>, <https://a360.co/3ZfB4rm>


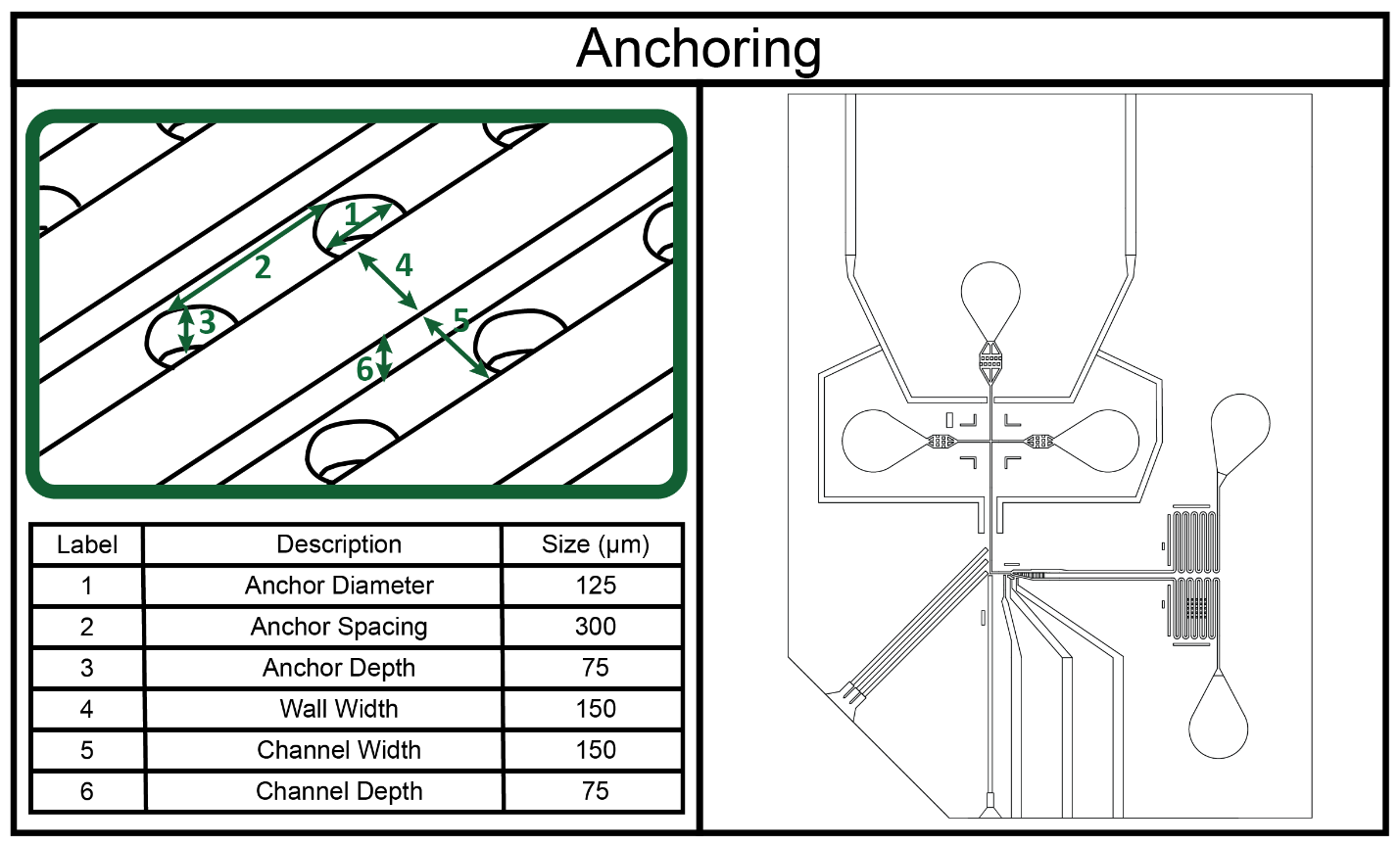


**Supplemental Figure 10: Data sheet for anchoring.** Fusion 360 Link: <https://a360.co/48cEeAe>


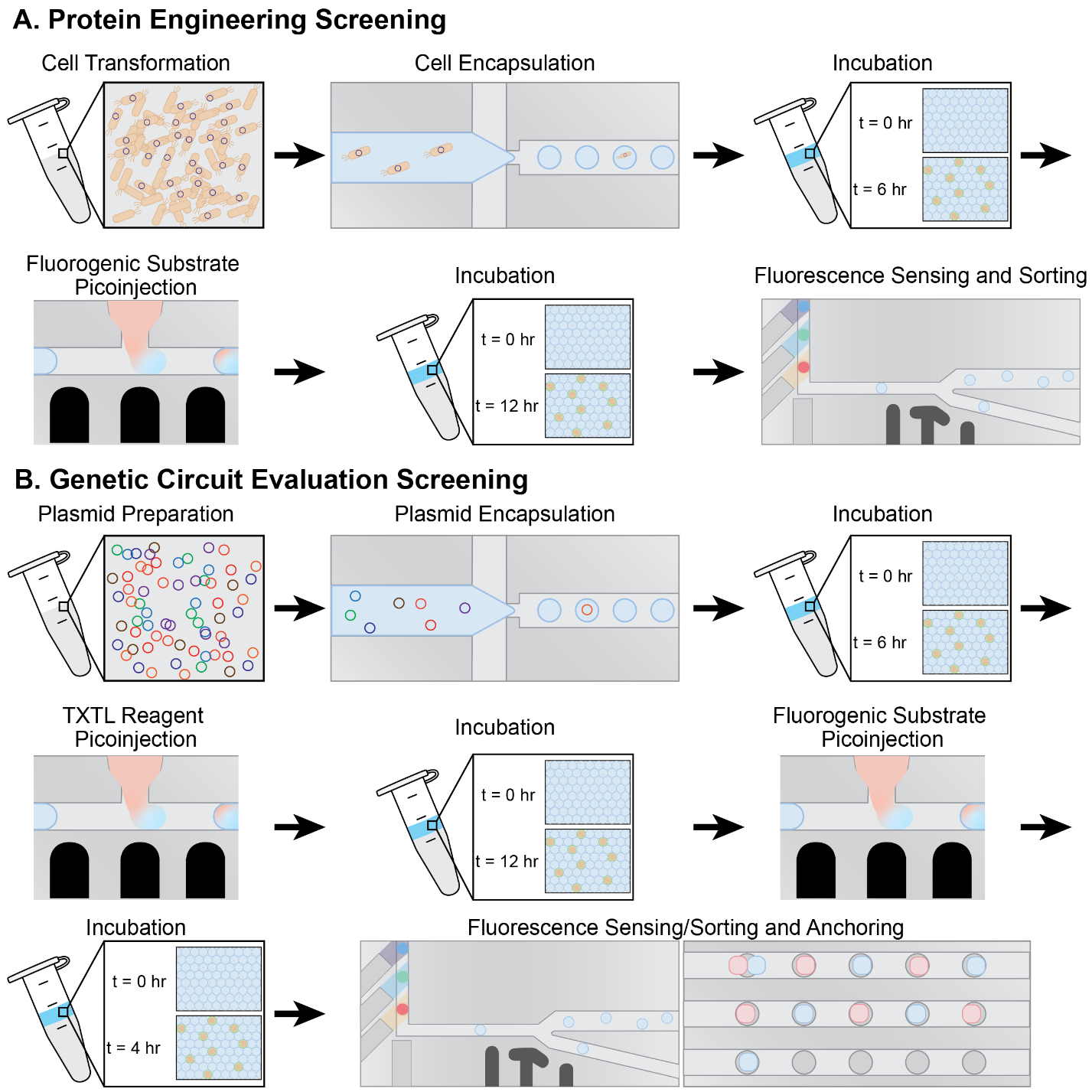


**Supplemental Figure 11: Microfluidic process for biological assays.** (A) Microfluidic pipeline for screening protein engineering constructs, including transformation of genetic library into cells, cell encapsulation in droplets, incubation, picoinjection of fluorogenic substrate, incubation of fluorogenic substrate and cells in droplets, fluorescence sensing and sorting, and sequencing of positively sorted droplets. (B) Microfluidic pipeline for screening cell-free genetic circuits, including plasmid encapsulation in droplets with rolling circle amplification mix, incubation, picoinjection of in vitro transcription and translation reagent, picoinjection of fluorogenic substrate, and fluorescence sensing and sorting into anchor grid.

**Supplementary Videos:**

**Supplementary Video 1: 2023_anchoring_200FPS_slowed_to_25FPS.mov**

Video of blue and red droplet reinjection and anchoring. Video was captured at 200 FPS and slowed down to 25 FPS in supplemental video.

**Supplementary Video 2: 2023_reinjection_400FPS_slowed_to_25FPS.mov**

Video of yellow and blue droplet reinjection. Video was captured at 400 FPS and slowed down to 25 FPS in supplemental video.

**Supplementary Video 3: 2023_single_channel_fluorescence_sensing_400FPS_slowed_to_25FPS.mov**

Video of single channel fluorescence sensing of yellow droplets. Video was captured at 400 FPS and slowed down to 25 FPS in supplemental video.

**Supplementary Video 4: 2023_sorting_386FPS_slowed_to_25FPS.mov**

Video of yellow and blue droplet sorting. Video was captured at 386 FPS and slowed down to 25 FPS in supplemental video.

**Supplementary Video 5: 2023_software_tutorial.mp4**

Software tutorial video explaining how to use image software.

**Supplementary Files:**

**Supplementary File 1: 2023_droplet_generator.f3d**

Fusion 360 file for droplet generation.

**Supplementary File 2: 2023_picoinjector.f3d**

Fusion 360 file for droplet picoinjection.

**Supplementary File 3: 2023_single_channel_sorter.f3d**

Fusion 360 file for single channel fluorescence sensing and sorting.

**Supplementary File 4: 2023_triple_channel_sorter.f3d**

Fusion 360 file for triple channel fluorescence sensing and sorting.

**Supplementary File 5: 2023_single_channel_fluorescence_sensing.f3d**

Fusion 360 file for single channel fluorescence sensing.

**Supplementary File 6: 2023_triple_channel_fluorescence_sensing.f3d**

Fusion 360 file for triple channel fluorescence sensing.

**Supplementary File 7: 2023_anchoring.f3d**

Fusion 360 file for triple channel fluorescence sensing, sorting and anchoring.

**Supplementary File 8: 2024_supplmentary_note_3_data_analysis.xlsx**

Spreadsheet for data analysis of biological examples in Supplementary Note 3.

**References**

1. Collins, D. J., Neild, A., deMello, A., Liu, A. Q. & Ai, Y. The Poisson distribution and beyond: Methods for microfluidic droplet production and single cell encapsulation. *Lab on a Chip* vol. 15 3439–3459 Preprint at https://doi.org/10.1039/c5lc00614g (2015).
2. Schoeman, R. M., Kemna, E. W. M., Wolbers, F. & van den Berg, A. High-throughput deterministic single-cell encapsulation and droplet pairing, fusion, and shrinkage in a single microfluidic device. *Electrophoresis* **35**, 385–392 (2014).
3. Kemna, E. W. M. *et al.* High-yield cell ordering and deterministic cell-in-droplet encapsulation using Dean flow in a curved microchannel. *Lab Chip* **12**, 2881–2887 (2012).
4. Park, S. Y., Wu, T. H., Chen, Y., Teitell, M. A. & Chiou, P. Y. High-speed droplet generation on demand driven by pulse laser-induced cavitation. in *Lab on a Chip* vol. 11 1010–1012 (Royal Society of Chemistry, 2011).
5. Lashkaripour, A., Silva, R. & Densmore, D. Desktop micromilled microfluidics. *Microfluid Nanofluidics* **22**, (2018).
6. Holstein, J. M., Gylstorff, C. & Hollfelder, F. Cell-free Directed Evolution of a Protease in Microdroplets at Ultrahigh Throughput. *ACS Synthetic Biology* **10**, 252–257 (2021).
